# Striking a Balance: Mitigating Fraud While Ensuring Equity in Online Qualitative Research Recruitment

**DOI:** 10.2196/68393

**Published:** 2025-08-27

**Authors:** Eunji Cho, Laura F Lewis, Elizabeth G Broden Arciprete

**Affiliations:** 1 Connell School of Nursing Boston College Chestnut Hill, MA United States; 2 College of Nursing and Health Sciences University of Vermont Burlington, VT United States; 3 School of Medicine and School of Public Health Yale University New Haven, CT United States

**Keywords:** online recruitment, social media recruitment, qualitative research, fraudulent participants, data integrity, participant inclusivity

## Abstract

After the COVID-19 pandemic, online recruitment became a critical component of qualitative research in health care fields. However, fraudulent participants targeting research incentives have become more prevalent in health studies, raising significant issues for research ethics, data integrity, and the inclusion of diverse patient voices. While qualitative health research aims to listen to and amplify patients’ and communities’ voices, such fraud can severely impact research quality and foster mistrust toward participants. This issue is particularly critical in qualitative studies, where careful communication, engagement, and mutual trust between researchers and participants are hallmarks of the research process, especially when working with populations considered marginalized. Behaviors that researchers may associate with fraudulent participants also appear in the communication patterns of groups considered marginalized, especially when discussing sensitive topics. This similarity could lead to misplaced suspicion, unintentionally disadvantaging populations considered marginalized when they attempt to share their experiences. In this paper, 3 qualitative nursing researchers reflect on their experiences with recruitment and data collection in recent studies and provide recommendations based on their experiences and a review of relevant literature. These include methods for addressing challenges related to potentially fraudulent participants and balancing ethical approaches with justice and inclusivity while preserving research integrity, drawing on existing strategies from previous studies facing similar issues. The paper also identifies unaddressed areas requiring future attention and highlights the importance of promoting inclusivity for diverse populations and populations considered marginalized who may be disproportionately affected by mistrust in participant integrity.

## Introduction

### Background

Online recruitment has become an essential strategy in health care research involving human participants, especially for engaging diverse populations that are traditionally underrepresented in health research. Researchers frequently identify target populations using social media platforms (eg, Facebook, Instagram, Reddit, and Craigslist) and websites popular among specific groups [1,2], as well as ResearchMatch, a national registry for clinical research volunteers [3]. These methods help overcome physical barriers such as location and time, facilitating cost-effective access to large pools of potential participants [2]. Online recruitment approaches are especially effective for recruiting hard-to-reach groups, such as minoritized and rural populations, which are often underrepresented in health-related studies [4]. The importance of these strategies was highlighted during the COVID-19 pandemic, when traditional recruitment methods were limited.

Despite its benefits, online and social media recruitment poses significant risks, such as the potential for fraudulent activities that threaten research integrity [5]. For instance, Pozzar et al [6] found that 94.5% of their online survey responses completed within 7 hours were fraudulent. Participants may use fake identities, complete surveys multiple times, or use bots or server farms to maximize financial incentives [7]. To mitigate these risks, researchers suggest incorporating technologies such reCAPTCHA, filtering IP addresses, and adding qualitative components such as free-text responses and interviews [8,9]. However, we found unique patterns in fraudulent activities within online qualitative research that could not be prevented by strategies typically used in other types of research, highlighting the need for more tailored and specific approaches.

### Real-World Experiences and Emerging Patterns

To explore real-world challenges in online qualitative research recruitment, the 3 authors of this paper shared their individual experiences and identified common patterns of participant fraud. Each author encountered suspicious activity following social media recruitment, including a sudden surge of individuals expressing interest and completing eligibility questionnaires within a short time frame, often using oddly similar email formats, and providing inconsistent or implausible participant information. For example, some claimed a demographic profile rarely represented in such studies or reported an age that was later revealed to be significantly different during the screening process. Many fraudulent participants demonstrated limited understanding of the study topic, gave vague or repetitive answers, avoided meaningful interaction, and appeared primarily motivated by compensation. In response, the authors implemented stricter verification procedures and revised recruitment and consent protocols. However, this process caused significant stress and uncertainty for the research teams.

We described each of our experiences in Multimedia Appendix 1 due to the length of the content. We highly recommend that readers, especially those who are relatively new to qualitative research, review our experiences before examining our recommended strategies, as understanding the context would greatly help in recognizing emerging patterns of fraudulent participants in online qualitative research. We then searched the existing literature to identify and summarize similar recruitment challenges and strategies to prevent potentially fraudulent activities at each research phase, from preparation to dissemination. While we did not limit the time range of the search, we found that most studies were published during or after the COVID-19 pandemic, from 2021 to 2024. We excluded survey-only studies because the strategies used to prevent fraud in those studies focus on bots and hacking, which involve different challenges than those in online qualitative research.

This viewpoint paper was shaped by our commitment to promoting both rigor and equity in qualitative research. While we provided a list of prevention strategies, we argue that such strategies must not only be effective but also ethically justified and inclusive, particularly because online research spaces can amplify existing disparities in access, trust, and representation.

## Methodological, Practical, and Ethical Considerations

### Overview

On the basis of our experience (Multimedia Appendix 1) and a literature review (Multimedia Appendix 2 [1,5,7,10-22]), we suggest strategies for each phase of the qualitative research process to help prevent fraudulent participation in online studies. We focus on methodological, practical, and ethical considerations that protect participants’ rights and uphold research integrity. We searched the literature using databases such as CINAHL, PubMed, and Scopus, and also applied a snowballing approach by reviewing reference lists of relevant articles. NVivo 15 (Lumivero) was used to organize and code the reviewed literature, allowing us to identify recurring themes and refine our recommendations.

In this paper, we included a condensed version of the table contents from Multimedia Appendix 2 (Textbox 1), but we also highly recommend reviewing the details in Multimedia Appendix 2, as it presents a series of reflection questions, rationales, strategies, and ethical and inclusive considerations to help prevent the false exclusion of genuine participants during fraud prevention efforts and to ensure basic respect for everyone involved, regardless of their intent. We hope that this information can work as a step-by-step guide for all level researchers who are planning a qualitative study using online recruitment strategies.

**Fraud moderation strategies across phases of online qualitative research.**

**Design and planning**

- Engage in early reflection to understand the target population’s characteristics and needs, preparing to recognize unusual patterns that may indicate potential fraud without misinterpretation.
- Train and emotionally prepare the research team to critically assess the authenticity of participant responses without bias or hostility.
- Develop and pilot-test screening criteria tailored to the population’s characteristics (eg, cognitive level and literacy) to ensure accuracy and anticipate challenges.
- Offer screening and interview questions that align with the knowledge and lived experiences unique to the target population.
- Involve community representatives and the institutional review board (IRB) in developing fraud prevention strategies to enhance validity and ethical sensitivity.
- Ensure that all fraud prevention strategies are reviewed for ethical appropriateness and compliance with relevant regulations, and that they do not disproportionately burden or exclude groups considered historically marginalized.
- Plan for handling compensation of potentially fraudulent participants. Ethical dilemmas may arise when compensating participants whose authenticity is in question. Providing compensation could risk reinforcing fraudulent behavior, yet these individuals may still have contributed time and effort. Consider options, such as prorated compensation based on the level of participation, and document team decisions transparently. Engage IRBs or funders early to develop an ethically justified and context-appropriate approach.

**Recruitment and enrollment**

- Develop recruitment strategies that are intentionally inclusive and designed to engage diverse populations in accessible ways.
- Clearly explain the study’s purpose to support participants’ understanding of their role, while limiting eligibility details in advertisements to essential information only, to prevent fraudulent participants from tailoring their identity to meet the eligibility criteria.
- Use unique links for different recruitment sources to monitor and disable links if fraud is detected.
- Use incentives that are ethically appropriate, culturally relevant, and appealing to the target population to encourage participation while minimizing coercion.
- Develop a team-based plan to review and discuss potentially suspicious responses in emotionally, ethically, and methodologically informed ways.
- Ensure that recruitment materials are accessible and understandable to the target population.
- To reduce the risk of fraudulent participation, describe monetary incentives in general terms in recruitment materials, and disclose detailed incentive requirements after participants have been confirmed and enrolled.
- Implement both automated and manual (eg, phone or video call) procedures for verifying participant information, while ensuring fairness and minimizing harm.
- Use open-ended screening questions that assess topic-specific knowledge or lived experience, without compromising the privacy or dignity of potential participants.
- Provide a platform for potential participants to ask questions and receive accurate, timely responses.
- Develop a monitoring dashboard to detect unusual activity collaboratively as a team, even when some members are not directly involved in recruitment (eg, a sudden surge in responses at atypical times).
- Document potential fraud indicators using both reflective judgment and systematic reasoning within the team.
- Observe the tone and content of participant communication. Flag responses that appear disengaged, overly general, or primarily focused on compensation, as these may indicate a lack of relevant experience or genuine interest in the study topic.

**Data collection, analysis, reporting**

- Apply consistent data collection methods to maintain reliability and validity.
- Train team members involved in data collection to recognize and appropriately follow up on brief or disengaged responses, such as by probing for clarification or noting signs of disengagement, while avoiding bias and ensuring accurate reporting of observations.
- Develop standardized scripts and classification systems to address concerns in real time, prioritizing participant dignity; use indirect explanations (eg, technical difficulties) only when direct disclosure may cause harm.
- Document and review suspicious cases with the research team rather than relying on individual judgment.
- Implement technical measures (eg, CAPTCHA [a completely automated public Turing test to tell computers and humans apart]) to support data authenticity and mitigate automated or fraudulent responses.
- Maintain data integrity and confidentiality throughout analysis to protect participants’ information.
- Establish a clear, team-driven process for categorizing, retaining, reporting, or discarding data suspected to be fraudulent, with the option to offer prorated compensation based on partial participation.
- Clearly report how such data were handled, including the rationale for classifying certain cases as suspicious.
- Report findings transparently, acknowledging limitations, and including any threats to validity posed by potential fraud.

Table 1 lists the articles we reviewed for this section. We also included the field of research for each article, showing that fraudulent activities can occur not only in health research but across many disciplines. Some literature suggests that health research may be relatively safer from fraud because it often focuses on specific medical conditions, which could serve as a barrier for fraudulent participants, as they would need more specific knowledge [10]. However, many health studies, including ours, have reported encounters with fraudulent participants who falsely presented themselves as patients without any relevant knowledge and provided irrelevant responses, potentially compromising data integrity. While we summarize both our recommendations and lessons from the existing literature, we encourage readers to consult these references for further guidance in their future work.

**Table 1:** List of peer-reviewed articles on fraudulent participation in online qualitative research across disciplines (2021-2024).

Study	Title	Study population and characteristics	Location and time frame	Field and study method
Glazer et al [5]^a^, 2021	Liar! Liar! Identifying eligibility fraud by applicants in digital health research	Older adults with insomnia; recruited via flyers, health care provider referrals, online advertisements, and social media	United States; participants recruited over 62 weeks	Health sciences; online intervention with phone screening
Roehl and Harland [13], 2022	Imposter participants: Overcoming methodological challenges related to balancing participant privacy with data quality when using online recruitment and data collection	Adults working with students in grades 6-12 (eg, educators, media specialists, and coaches); recruited via a social media professional learning network and a university’s participant pool	Location not specified; study conducted during the COVID-19 pandemic	Education; qualitative interviews via Zoom or phone
Davies et al [12]^a^, 2023	Management of fraudulent participants in online research: Practical recommendations from a randomized controlled feasibility trial	Adolescents and young adults (aged 16-25 years) with eating problems; recruited via websites, social media, school announcements, and clinicians	United Kingdom; 4-week intervention conducted in 2023	Health sciences; online randomized controlled feasibility trial
Mizerek et al [1], 2023	Identifying and mitigating fraud when using social media for research recruitment	Emergency nurses and online research participants recruited via social media surveys	United States; time frame not reported	Health sciences; virtual semistructured Zoom interviews
Owens [10], 2023	Encountering deception in virtual spaces: guidelines for virtual ethnography	Individual claiming to live in New York City public housing; recruited via Craigslist	United States; time frame not reported	Sociology; email communication, an interview, and a reflective follow-up with a suspected fraudulent participant
Sefcik et al [11], 2023	When snowball sampling leads to an avalanche of fraudulent participants in qualitative research	Caregivers of individuals with Alzheimer disease or related dementias and chronic wounds; recruited via ResearchMatch	United States; time frame not reported	Health sciences; internet-based semistructured interviews
Willis et al [7]^a^, 2023	The detection and management of attempted fraud during an online randomised trial	Clinicians and Managers affiliated with 5 United Kingdom national clinical audit programs; recruited via email invitations sent through audit program networks	United Kingdom; studied in 2019	Health sciences; online fractional factorial randomized trial with self-administered feedback review and questionnaire
Carey et al [21], 2024	Methods to reduce fraudulent participation and highlight autistic voices in research	Autistic individuals from underrepresented groups; recruited via institutional and community listserves	United States; recruitment began in late 2023	Health sciences; online focus groups
Kumarasamy et al [20], 2024	Evaluating the problem of fraudulent participants in health care research: Multimethod pilot study	Health care researchers with experience in encountering fraudulent participants; recruited via purposive and snowball sampling through institutional listserves	Canada; time frame not reported	Health sciences; REDCap survey and Zoom-based semistructured interviews
McLachlan et al [19], 2024	Fraudulent participation in psychological research using virtual synchronous interviews: ethical challenges and potential solutions	Parents of children and youth with pain or neurodevelopmental conditions; recruited via social media and research participant platforms	Canada; conducted during the COVID-19 pandemic	Health sciences and psychology; virtual synchronous interviews
Mistry et al [18], 2024	Fraudulent participation in online qualitative studies: Practical recommendations on an emerging phenomenon	Participants across 3 qualitative studies (individuals with skin cancer, adult women, and patients with advanced cancer); recruited via social media, support groups, and paid online advertisements	United Kingdom; study dates not reported	Health sciences; qualitative interviews and online focus groups
Panicker et al [17], 2024	Understanding fraudulence in online qualitative studies: From the researcher’s perspective	US-based qualitative researchers in the human-computer interaction field; recruited via online flyer and research network	United States; time frame not reported	Social sciences and human-computer interaction; online semistructured interviews
Pellicano et al [22], 2024	Letter to the editor: A possible threat to data integrity for online qualitative autism research	Autistic individuals in 3 online qualitative studies; recruited via social media and community partners	Australia, United Kingdom, and United States; time frame not reported	Health sciences and psychology; online qualitative interviews
Pullen Sansfaçon et al [14], 2024	Dealing with scam in online qualitative research: Strategies and ethical considerations	Adolescents and young adults (aged 15-25 years) who have discontinued gender transitions; recruited via purposive and snowball sampling through social media platforms	International; 2 recruitment rounds in fall 2020 and winter 2022	Social work; in-depth individual interviews conducted online
Wang [15], 2024	Do participants lie? Imposter participants in online qualitative research	US nationals living and working in China as English teachers or migrant workers; recruited via social media job information groups	United States and China; study conducted during the COVID-19 pandemic	Language and literacy education; digital ethnography with qualitative Zoom interviews
Wright et al [16], 2024	Participant fraud in virtual qualitative substance use research: Recommendations and considerations for detection and prevention based on a case study	Individuals with cannabis-related problems; recruited online and through hospitals, clinics, and community settings	Canada; time frame not reported	Health sciences; online qualitative interviews

^a^These 3 studies are not fully qualitative in design. However, they were included because they focus on analyzing patterns of fraudulent participation and propose practical, transferable strategies that are highly relevant to and can inform qualitative research practice.

### Phase 1: Study Design and Preparation Phase

Before the study begins, researchers should engage in early preparation by reflecting deeply on their target population and research topics to understand the context, characteristics, needs, and unique risks of the population, including the potential for deception. Unexpected responses may be observed not only among fraudulent participants but also among authentic participants who have diverse language backgrounds, cultures, or communication styles [21].

In addition to understanding the population, researchers should reflect on their own methodological and emotional preparedness for handling fraud and data integrity issues, as well as how their study’s goals and epistemological framework may shape appropriate fraud prevention strategies. Many researchers, including the authors of this study, have shared their fear and concern about data integrity upon realizing that some participants had falsified their identities [14,15,18,19]. Anticipating and preparing for such challenges, while having a clear sense of direction in their research, can help researchers respond more promptly and confidently when these issues arise.

During the study design and preparation phase, researchers could incorporate specific strategies into their protocol to detect and prevent fraud across all stages of the research process. Research teams may consider allocating sufficient time, funding, and staffing resources to support participant verification and ongoing monitoring. In addition, researchers could thoughtfully integrate technological tools with a clear understanding of their strengths and limitations. For example, several researchers recommended checking IP addresses to verify whether participants’ disclosed locations match their actual geographic locations but also highlighted that the use of shared or public internet networks or intentional masking of IP addresses could lead to misinformation [12,13,19,21]. No single technological tool can perfectly prevent fraudulent activity, which underscores the need for greater attention and vigilance from researchers.

Developing a clear and coordinated communication plan is also critical to ensure timely responses to emerging issues, including suspected fraud. Some literature suggested maintaining a respectful tone when responding to suspected fraudulent participants by thanking them for their interest but informing them that they cannot proceed, using nonconfrontational explanations such as an overwhelming number of participants or ineligibility identified during the screening process [7,13-15]. Most researchers emphasized that maintaining a professional tone is important, even when dealing with clearly fraudulent participants, and that a well-prepared protocol can help guide professional responses and attitudes, even in the face of unexpected and potentially distressing experiences for researchers.

During recruitment preparation, researchers could consider using closed or verified recruitment channels to minimize exposure to fraudulent participants, although this approach may introduce additional challenges by limiting access to potential participants [10,16,17,20]. To identify the most appropriate channels, researchers may need to invest sufficient time and effort to understand the platforms and build necessary connections, if needed. Recruitment materials could balance the need to communicate clear eligibility criteria while protecting against potential manipulation, as some participants may tailor their identities to pass screening [13,18-20]. Incentives could be selected and delivered carefully, with attention to fraud prevention, participant accessibility, and ethical fairness [5,12,17,19]. To minimize manipulation, it can be helpful to limit the visibility of incentives in recruitment materials while still ensuring ethical transparency [12,16,20,21]. Researchers might implement additional screening or verification processes to confirm participant authenticity [5,16,21,22]. Informed consent forms should clearly communicate participation requirements, technological expectations, ineligibility criteria, and procedures for handling suspected fraud [11,15,16,19]. Finally, it may be beneficial to include fraud prevention strategies in institutional review board (IRB) submissions and to discuss potential scenarios to ensure appropriate oversight and preparedness.

Throughout all these processes, engaging mentors, IRBs, funders, and other support systems early in the preparation phase is important for collaboratively establishing fraud management strategies [17-19]. By involving these support systems and all team members, researchers can develop a flexible, systematic decision-making process for detecting, assessing, and responding to fraud throughout the study. At the same time, it is important to prioritize fraud prevention while respecting participant dignity, privacy, and equitable access. Researchers are advised to avoid assumptions based on demographic characteristics, culture, language, or communication style, and to use transparent and proportionate verification methods approved by the IRB. Embedding ethical reflection into team practices may further support responsible research conduct. Rather than viewing all participants with suspicion, researchers are encouraged to build engagement strategies that foster trust while remaining alert to potentially fraudulent activity.

### Phase 2: Recruitment and Screening Phase

Once researchers ensure that they are well-prepared with ethical protocols, support systems, and awareness to manage both expected and unexpected challenges, including fraud, they should apply concrete strategies and use reflexive practices, such as research memoing, to guide sound decision-making [22]. Most importantly, they are encouraged to anticipate and consider potential burdens when planning study timelines and allocating resources during this phase. It is important to build rapport with participants while staying alert to possible signs of fraud, supported by clear protocols for managing unexpected events. Ongoing monitoring of participants’ responses and expressions of interest may be more demanding than initially expected, so research teams are advised to dedicate sufficient effort to this continuous task.

Fraud detection strategies can benefit from a combination of automatic and manual systems. Researchers are advised to monitor for sudden influxes of responses, especially during unusual hours or from participants whose claimed time zones do not match their submission patterns [7,14,21]. Authentication tools such as IP tracking, time zone checks, CAPTCHA (a completely automated public Turing test to tell computers and humans apart), and geolocation cross-checks may support location verification, although they require IRB approval and each comes with limitations [7,15,16]. Monitoring suspicious email domain types and patterns (eg, full name plus random numbers@gmail.com) and checking for duplicate information across submissions can help flag suspicious participants, although reliance on these indicators alone is not recommended [14,19]. When feasible, researchers may collaborate with IT specialists to analyze submission patterns and log completion times for each survey to detect unusually short durations [14].

Screening processes could include open-ended questions that assess topic-specific knowledge and lived experience, attention-check items, and layered verification steps such as preinterview phone or video screening [12,14,18,20]. Screening responses should be reviewed for appropriateness, relevance, and consistency with later communications or interviews, as fraudulent participants often do not remember their previous answers or show a lack of understanding of the research topic. Monitoring participant communication tone is important, with attention to overly vague, overly scripted, disinterested in the interview, or incentive-focused responses, while maintaining a respectful and nonthreatening approach to verification [13,14,18]. When requesting additional materials, such as diagnosis forms (for studies targeting patient populations) or professional profiles (for studies targeting professionals), researchers are encouraged to clearly explain the purpose, obtain participant consent, establish strategies for either secure storage or immediate disposal after verification, and ensure that all procedures are approved by the IRB [5,10,18,21].

Video confirmation can be one part of participant validation but cannot be the sole determinant of eligibility. Expectations around camera use are ideally stated in the consent process, with flexible options, such as brief camera activation or blurred backgrounds, offered to participants [11,16,20,21]. It is also helpful to track recruitment sources systematically, for example, by assigning different survey links to different channels, which can support rapid monitoring and adjustment if needed [11,16,17].

Finally, a team-based, systematic decision-making process to validate participant authenticity could be applied throughout the recruitment process [14,15,17,21]. Staff recruiting participants should be trained to identify potential red flags, document unclear responses, and discuss concerns with the team [14]. Multi-indicator approaches, such as combining response quality, time zone checks, and video confirmation, could guide fraud detection efforts. Several researchers recommended that a tiered flagging system (eg, color labeling categorized as fraudulent, suspicious, or authentic) can help prioritize cases for review [21]. Teams are encouraged to develop a shared understanding that this process can be emotionally burdensome and confusing, as it involves following well-prepared criteria and strategies while also, at times, relying on personal judgment and instincts [14]. Regular team discussions, grounded in open-mindedness and evidence-based comparisons to previous case examples, could guide final decisions. Combining both automatic tools and manual review strengthens the integrity of participant validation while maintaining respect for participant dignity, privacy, and inclusion.

### Phase 3: Data Collection, Analysis, and Dissemination Phase

Even when rigorous screening processes are applied during recruitment, researchers may still encounter suspicion during or after data collection and experience frustration, feeling that their data have been compromised. However, multiple strategies have been proposed to help promote data integrity while encouraging researchers to transparently report their experiences in scientific documents, fostering healthy discussions and collective efforts to minimize fraudulent activities within the qualitative health research community.

During data collection, maintaining participant privacy while ensuring data integrity is critical. Sensitive information could be linked only to pseudonyms or nonsensitive identifiers, balancing traceability with confidentiality [7,13]. It is important to clearly define what identifying information is necessary, protect it appropriately, and apply systematic protocols for reviewing, retaining, or discarding potentially fraudulent data. Detection efforts could be strengthened when initiated early: team members can be trained to recognize early indicators of fraud using both structured reasoning and instinct, supported by concrete examples of red flags during onboarding [14,22]. Observations and concerns could be documented consistently during data collection, using reflexive journals and regular team discussions to guide decisions when uncertainties arise [22].

Researchers may consider layering verification strategies into the data collection process to identify inconsistencies and enhance credibility. Incorporating repeated key questions, cross-checking screening and interview responses, and using factual or lived experience questions can help reveal inconsistencies [11,18,20,22]. Major discrepancies are better addressed through team discussions rather than immediate exclusion of participants [21,22]. Interviews can be structured with flexibility, allowing follow-up prompts to clarify brief or vague responses. When appropriate and IRB-approved, researchers may consider asking participants to briefly turn on their cameras or verify location-related information, while carefully noting any mismatches between verbal claims and visual or auditory cues [14,16].

A respectful, prepared script could guide real-time responses if fraud is suspected, allowing interviews to pause or end politely while preserving participant dignity [12]. In rare cases where researchers determine that transparent disclosure could cause more harm than good, they may choose to end the interview by citing a neutral reason, such as technical difficulties. However, this approach should be used with caution and not adopted as a standard practice. Emotional preparedness and access to team or institutional support are essential for handling these situations.

Initiating data analysis early and continuing it alongside data collection can help facilitate real-time detection of suspicious patterns [11,18,20,22]. Preliminary coding may include categories such as *suspicious responses*, and reflexive journals can be used to capture emotional reactions and document decision-making processes [11,18,20,22]. Analysts are encouraged to review qualitative responses carefully for coherence, depth, and alignment with the study’s focus, while remaining cautious of fabricated but detailed narratives. Monitoring response patterns across participants, including demographic similarities, voice, tone, and environmental cues, can also support systematic detection efforts [14]. Metadata such as IP addresses, email domains, and timestamps may further assist in identifying broader trends, although they should not serve as the sole basis for exclusion [14].

When discrepancies or concerns arise, team-based discussions, independent reviews (such as colistening to audio), and multi-indicator flagging systems can support more balanced and less biased decision-making [14,19,20]. It is advisable to align compensation procedures with verification efforts: researchers could implement a standard delay between data collection and payment, use verification methods if necessary, and honor preapproved compensation policies [14,18]. Manual review of suspicious compensation requests, clear documentation of decisions, and transparency with IRBs could be critical steps in maintaining ethical standards. Follow-up contact with participants, member checking, and community engagement may also provide additional opportunities to validate data authenticity [13,16,22].

Dissemination practices should also reflect a commitment to transparency, dignity, and research integrity. Suspicious cases and the steps taken to address them can be documented thoroughly through memos, audit trails, and reflexive journals [22]. Publications and reports may include clear, neutral explanations of any data exclusion, using footnotes or methods sections to describe detection processes without stigmatizing participants [21,22]. When deciding what details to disclose publicly, researchers are encouraged to use careful judgment to avoid giving future fraudulent actors an advantage [5,21,22].

Experiences with fraud could be reported not only internally to IRBs, funders, and sponsors but also shared with the broader academic community [7,17,19]. Transparent discussions through publications, presentations, and policy dialogues can help build field-wide awareness and strengthen preparedness. Researchers are encouraged to collaborate proactively with IRBs in developing detection protocols, consent language, and reporting procedures [5,17,19,22]. Finally, ongoing engagement in cross-disciplinary conversations about emerging technological risks and opportunities, such as the use of artificial intelligence for both fraudulent participation and fraud detection, can contribute to the evolving standards of ethical qualitative research [19,22].

### Ethical and Inclusivity Considerations

When we encountered suspicious participants in our research without preparation, we experienced multiple emotions that left us confused and concerned. There was fear of losing data integrity, a sense of betrayal toward participants because qualitative research often involves building trust, and guilt over suspecting all participants during the research process, especially when they belong to populations considered vulnerable and marginalized. We also felt confusion, as we were unsure whether our feelings of suspicion stemmed from bias and assumptions or from logically acceptable reasons. Researchers with similar experiences note that such feelings are common and normal, but they can also significantly impact the research process and its outcomes [14,15,18,19].

Researchers working with populations considered vulnerable strongly recommend engaging these communities not just as participants but as coresearchers or partners throughout all phases of research, in ways that respect cultural, linguistic, cognitive, and privacy differences [12,20-22]. Their involvement can enhance both the accessibility and rigor of the study by helping develop screening questions, offering feedback on design features, and identifying nuanced cues of authenticity, such as specific language or insider knowledge, when reviewing potentially suspicious accounts. However, building trusting relationships with communities requires time, effort, and resources, including allocating time and funds to support community partners in verification and design processes. These contributions strengthen ethical decision-making but require preparation, mutual respect, and sustained commitment.

Fraud prevention efforts should not come at the cost of excluding or alienating authentic participants. Recruitment, screening, and communication strategies could be designed to promote accessibility and inclusion, ensuring that privacy-protective behaviors are not mistaken for fraud. For example, participants could be invited to turn on their camera briefly for verification and then turn it off for the remainder of the interview to reduce social discomfort. Other supportive strategies include offering a prerecorded video introduction of the researcher, allowing participants to choose from a diverse pool of interviewers, and providing flexible options for verifying identity. Researchers may also consider how compensation methods, such as requiring a physical address for gift cards, might inadvertently exclude or endanger participants who are unhoused or living in unsafe environments. Including flexibility in the compensation plan can help promote equitable participation.

Importantly, researchers are encouraged to avoid dismissing data as fraudulent solely because it diverges from the dominant narrative. This is particularly important when the data come from participants with marginalized or minoritized identities. In ambiguous cases, it could be helpful to examine whether any discomfort arises from bias or from verifiable inconsistencies, and to make efforts to validate divergent experiences rather than exclude them prematurely. Participants’ reluctance to comply with verification procedures may stem from privacy concerns unrelated to fraud, particularly among those from historically targeted groups. Protocols can acknowledge this possibility and include clear guidance for distinguishing between legitimate privacy behaviors and red flags for exclusion. For instance, researchers might request that participants disable virtual private networks during verification, but such requests should be communicated transparently and respectfully. These practices can help ensure that verification efforts remain both effective and ethically justified.

Clear and transparent consent processes are essential for building trust, and monitoring practices should be proportionate, ethically justified, and sensitive to the diverse realities of participants’ lives. While some argue that identified fraudulent participants should not be reimbursed even if they complete interviews, to avoid reinforcing their behavior, others suggest that ethical compensation may still be warranted for anyone who finishes their participation [14,18]. This dilemma highlights the need for ongoing ethical discussion and well-developed protocols to address such issues.

Embedding reflexive practices throughout the research process could be another strategy. Promoting ethical rigor requires integrating reflexive practices across all stages of recruitment, data collection, analysis, and reporting. Researchers could constantly question their assumptions, attend to emotional responses, and document decision-making processes through memos, team discussions, and structured reviews. Incorporating multiple perspectives and recognizing the limits of individual intuition can reduce bias and improve the fairness of participant validation. In this way, reflexivity can support both scientific credibility and ethical responsibility.

While dealing with fraudulent participants can be a challenging experience, it is essential to maintain professional and ethical standards in prevention and response. Such professionalism involves balancing vigilance with empathy and transparency. Fraud detection efforts are best grounded in clear, evidence-based systems rather than subjective judgment or stereotypes, and all participants should be treated with dignity, even when they appear suspicious. Decisions about eligibility, compensation, and data inclusion could be guided by collaboratively developed and IRB-approved procedures, with transparent documentation throughout. By approaching fraud detection as part of responsible research practice, rather than a punitive response, researchers can protect both participant rights and research integrity.

Finally, ongoing self-reflection and ethical learning were important lessons for us. Self-reflection is vital for sustaining ethical awareness in the face of complex challenges such as participant fraud. Researchers may benefit from acknowledging the emotional burden of encountering deception, staying open to learning from difficult experiences, and seeking support from institutional or peer networks when needed. Transparent documentation of challenges and lessons learned can strengthen future research practices and contribute to broader conversations about ethical standards in qualitative research. Ultimately, fostering a culture of humility, openness, and shared responsibility will be key to navigating these evolving challenges. Table 2 provides an overview of the key principles.

**Table 2:** Inclusivity principles for mitigating fraud while promoting equity in online qualitative research.

Inclusivity principle	Description
Include community partners in all phases of research when possible.	Include community partners on the research team, when possible, to ensure that the design is accessible and respectful. To build trusting and mutually beneficial relationships with community partners, researchers may need to commit sufficient time and effort to engage with the community and identify ways in which the collaboration can benefit both parties without being overly burdensome. Openly discuss with partners what they hope to gain from the partnership. Allocate time and funds for community partners to be involved in verification processes when possible. Community partners can assist in developing questions to use during the verification process and can provide insight on subtle and nuanced cues about authenticity when reviewing potentially suspicious accounts (eg, specific words or phrases used and insider knowledge).
Consider ways to support accessibility that do not jeopardize participant verification.	Specifically, consider allowing participants to turn on their cameras for initial verification, after which they can turn them off for the remainder of the interview. This approach may decrease social anxiety during the interview. In addition, creating a prerecorded video introduction of the researcher before the interview, offering a diverse group of interviewers, and inviting participants to select their preferred interviewer can further enhance accessibility. For compensation with physical gift cards, consider populations that may be affected by the requirement of a physical address (eg, those who are unhoused and those for whom “home” is not a safe location to disclose information about the study). Include flexibility in the compensation plan in case these situations arise.
Be careful not to dismiss negative cases as fraudulent just because they differ from other responses.	When considering data that is suspicious but not clearly fraudulent, be careful not to discount negative cases solely because they reflect an experience that differs from other accounts. This is particularly important when suspicious data come from participants who report an aspect of identity that represents a minority perspective within the sample. Be mindful of aspects of identity that may affect an experience.
Support participants in navigating their privacy concerns without compromising verification processes.	Recognize that participants may be reticent to participate in data verification processes for reasons unrelated to fraud, particularly those from targeted and minoritized groups. Discuss the rationale with participants, and if they are not willing or able to comply, prepare for this in your protocol (eg, reasons that are acceptable versus reasons that would be cause for exclusion). Recognize that some participants may opt to use a virtual private network for their own privacy protections, which may obscure their IP address and geolocation. Request that users turn these off during the participant verification process.
Use targeted recruitment approaches to directly access the population of interest when possible.	Specifically identify trusted community members who can support gaining entrée into the community, for example, social media influencers, group moderators, and admin. Consider ways to use the recruitment budget that can both target and potentially support the population of interest (eg, collaborating with nonprofit organizations for networking support in exchange for fees rather than spending funds on social media advertisements).

## Unresolved Issues and Calls to Action for Future Research

While we offer potential strategies to prevent and address fraudulent research participation, several issues remain unaddressed that future studies should consider, particularly to protect scientific integrity and ensure the dignity, privacy, and inclusivity of participants.

### Issue 1: How Do We Preserve the Critical Role of Trust in the Online Qualitative Research Process Amidst Concerns About Participant Integrity, Without Biasing Our Results?

A recurring challenge in qualitative research is maintaining trust in participants’ authenticity without introducing bias. The literature has noted that fraudulent participants often display certain characteristics, such as unclear audio, poor video quality, suspicious email formats, or demographic information that does not align with study expectations. These patterns are consistent with our own experiences. However, relying on such cues can introduce unintentional bias and may undermine the respect and mutual curiosity that are foundational to qualitative inquiry.

Some researchers have suggested offering incentives that can only be used within the target country, based on findings that fraudulent participants may access studies from outside the intended geographic area. However, this approach could also risk excluding legitimate participants from diverse or mobile backgrounds. Researchers must carefully reflect on whether their concerns are based on verifiable patterns of fraud or on personal assumptions. For instance, some behaviors, such as declining to turn on a camera, may raise suspicion, but they can also reflect legitimate needs to protect one’s privacy and feel comfortable when discussing sensitive or traumatic experiences. This highlights the importance of maintaining both ethical and inclusive recruitment practices.

Building a collaborative mindset with participants may also serve as a useful strategy. Many participants join qualitative studies to make positive changes, have their voices heard, and contribute to their communities. Fraudulent participation not only compromises research integrity but also harms the communities the research is intended to support. Researchers may consider transparently disclosing the potential presence of fraudulent participants, explaining why this is a concern for both researchers and participants, and inviting participants to cooperate with further screening steps or follow-up questions. This shared understanding may strengthen trust and reinforce a collective investment in the research process. However, there should be more discussion and validation of such strategies, as they may also influence the study itself, including participants’ responses, researchers’ interpretations, and their interaction.

### Issue 2: How Can We Balance Recruitment, Screening, and Privacy?

Researchers often face a dilemma: efforts to protect participants’ privacy and reduce their burden, such as allowing them to disable video cameras during interviews or reducing the amount of documentation required to prove their eligibility, can make the study vulnerable to fraudulent participants. Although enhanced screening measures, including ID verification or professional account checks, can mitigate fraud, they may also deter legitimate participants and burden small research teams. Sefcik et al [11] suggested that having 1 experienced interviewer can be effective in identifying potentially fraudulent behavior. However, this approach may not be suitable for all teams, depending on the scope of the research or team size. Researchers must balance recruiting diverse populations with ensuring data integrity. While clear communication, standardized protocols, and staff training have been recommended, more detailed, accessible guidelines are needed to help researchers manage ongoing recruitment challenges in online qualitative research.

### Issue 3: How Should the Research Community Address Fraud and Adapt to Evolving Challenges?

Detecting fraudulent participants often relies on an experienced researcher’s intuition, but not all teams have such resources that can lead to structured team communication systems and standardized reporting practices, which could strengthen research integrity. Collaboration, transparent reporting, the sharing of strategies, and appropriate mentoring within the qualitative research community can support researchers at various stages, particularly early career researchers. As technology evolves, fraudulent strategies will evolve as well, requiring collective vigilance, institutional support, and the development of verified and shareable technical tools. There is an urgent need for clearer protocols to identify, respond to, and report suspicious participants.

Although multiple strategies, such as two-step screening, snowball sampling, and ID or document checks, can reduce risk, none of these measures offer complete protection. Even after applying precautionary strategies following initial incidents, we continued to encounter fraudulent participants in later recruitment rounds. While snowballing recruitment from reliable personnel has been recommended, Sefcik et al [11] reported issues originating from such sampling methods. Similarly, although the two-step screening process is widely supported to verify participant authenticity, we found that it did not fully prevent fraudulent activity. Individuals were still able to manipulate the process by tailoring their responses to meet eligibility criteria, often exploiting the detailed information presented in the screening questionnaires. Moreover, research scam groups may discover and use new or previously unrecognized methods to create multiple online identities.

Consequently, all suggested strategies should be considered together for implementation in research projects, and researchers must be prepared to handle unforeseen threats effectively. Shared experiences and reflections indicate that there is no single perfect solution for preventing fraudulent activity in online qualitative research. Instead, ongoing discussion is needed on how to combine multiple strategies in a practical and structured manner throughout all stages of the research process. Fraudsters continually adapt, which calls for flexible and comprehensive approaches to safeguard the integrity of qualitative research. While no universal guideline currently exists for addressing fraudulent participation, it is essential to establish a group of well-prepared experts, implement thoughtful guidelines and policies, and ensure that both the research community and academic institutions are informed and actively engaged in efforts to prevent and recover from fraudulent activities.

While all the literature we reviewed transparently reported their approaches for the benefit of future researchers, no standard protocol or reporting structure exists, especially for ambiguous situations during sensitive, in-depth interviews. Clearer guidelines are needed, including professional and IRB-approved language, protocols for addressing suspicious participants in the midst of data collection, data management strategies, and reporting metrics. Future research should focus on developing such protocols to enhance data integrity and protect the participation of legitimate respondents.

As there are numerous unanswered questions essential for future qualitative studies that consider online recruitment, we recommend specific actions and urge researchers to collectively address these issues. First, informing research support groups, such as IRBs, academic institutions, administrators, professional organizations, and funding agencies, can enhance community awareness and foster a collective effort to mitigate threats to data integrity and study validity. Second, there should be a concerted effort in partnership with communities to develop standardized protocols, guidelines, and tools to address this issue, with regular updates to keep pace with rapid technological developments and evolving schemes that exploit research intended to benefit human health and well-being. Furthermore, other institutions involved in research, such as academic journals and publication authorities, should recognize this issue and prepare tools such as standardized checklists, which can serve as research guidelines and promote transparent reporting by researchers as they disseminate their findings.

We strongly advocate for a research culture that accepts mistakes, failures, and unexpected outcomes related to these issues, as this can encourage researchers to report challenges more openly and provide valuable guidance to others. While researchers are expected to maintain transparency in reporting, there should also be editorial openness to publishing studies that honestly document and address fraudulent participation, rather than treating them as failed studies. Educators who teach research should also include information on rigorous research conduct and discuss strategies with their students to prevent such problems. Finally, there is an urgent need to build a community for collective action to tackle current problems and establish a sustainable task force that can continuously address evolving research scams targeting researchers.

We acknowledge several limitations in this paper, as it is not a systematic review but rather an effort to share our experiences and reflections and to raise urgent awareness of an issue that continues to affect many areas of research. Future work that focuses specifically on online recruitment in qualitative research across diverse settings, using more systematic approaches and formal evaluations, could further advance this area. While we have shared our recommendations on ethical and inclusivity considerations based on our experience and existing literature, we recognize that this remains an early stage of development and that more structured guidelines are needed, particularly given the complexity of the issue. Finally, we were not able to address the decision-making processes that might lead researchers to avoid online recruitment when the risks may outweigh the benefits. This will be an important area for future exploration.

We hope that, despite these limitations, our study can contribute meaningfully to advancing the field by offering practical strategies, highlighting this critical issue that requires further attention, and encouraging collective dialogue on the ethical and methodological challenges of online qualitative research.

## Conclusions

Online recruitment has proven to be an effective tool for engaging diverse population groups that are traditionally difficult to access through conventional, in-person strategies. This method allows participants to share their stories from the safety and convenience of their own environments, facilitating the inclusion of hard-to-reach populations and populations considered vulnerable in research. Despite these advantages, our exploration of the potential risks associated with online recruitment for qualitative studies—drawn from our experiences and those of other researchers—highlights significant challenges.

Throughout the research and preparation informing this paper, we recognized recurring patterns of fraudulent activity in our and others’ studies, particularly during or after the COVID-19 pandemic. This realization prompted a deeper examination of the literature and a reevaluation of past strategies aimed at preventing such issues. While techniques such as improving the clarity of research advertisements and informed consent forms, and implementing technical strategies to screen participants, have been used, they are often reactive rather than proactive, leading to varied threats to data integrity and study validity.

As qualitative researchers, both our experiences and the existing literature highlight the need for ongoing vigilance and innovative strategies to safeguard research integrity. The evolving nature of fraudulent participation in online studies calls for collective efforts within the research community to develop more effective approaches that manage risk while promoting equitable and inclusive practices. Because no single method offers complete protection, researchers must adopt flexible, multifaceted strategies grounded in ethical principles, institutional support, and meaningful collaboration with participants and communities. To protect data quality and participant trust, the field urgently requires standardized protocols, clear reporting guidelines, and a culture that values transparency, shared learning, and coordinated action.

## Appendix

Multimedia Appendix 1 Reflections on experiences.

Multimedia Appendix 2 Fraud mitigation strategies and inclusivity principles.

## Abbreviations

CAPTCHA: completely automated public Turing test to tell computers and humans apart
IRB: institutional review board
