# Supplementary material for: Striking a Balance: Mitigating Fraud While Ensuring Equity in Online Qualitative Research Recruitment
Source: J Med Internet Res. 2025 Aug 27;27:e68393. doi: 10.2196/68393 (PMC12423605; doi:10.2196/68393)
Supplement: Multimedia Appendix 1 [file jmir_v27i1e68393_app1.docx]

Appendix 1. Reflections on Experiences

| **Reflection 1 (EB):**  My team and I recently completed a qualitative study that explored how pediatric intensive care unit nurses identify, assess, and attend to suffering among dying children and their families. We recruited nurse participants from around the country using a variety of methods, including professional association networks, email, word of mouth, and social media. Anticipating potential issues with bot intrusion, we implemented CAPTCHA. However, we did not anticipate encountering fraudulent participants. We did not expect that people would lie about being a nurse on our eligibility screen in order to participate in an hour-long conversation about children’s suffering at end-of-life.  A handful of hints revealed that the participants who came to one of our focus groups were not in fact legitimately eligible for our study. First, we posted the study to Twitter (now X) and within hours saw a steep uptick in screenings. Our screening form incorporated some free text questions that sough insider knowledge relevant to the study (e.g. “Do you serve in any advanced roles on your unit?”, anticipated responses included charge nurse, preceptor, etc.). Among the responses following the Twitter posts, many of the free text responses were suspicious (e.g. nonsensical, illogical).  The second hint that made me suspicious that the respondents were not truly eligible for the study was patterns in contact information and demographic characteristics. Almost all of the respondents to the Twitter post had an email address in the same format (name123@gmail.com). Additionally, the demographic characteristics of these suspicious respondents did not match the pediatric ICU nursing workforce, which is predominantly White women.  Mindful that my suspicions were just that, suspicions, I took a conservative approach to these respondents. After careful discussion with my mentorship team, I marked participants that I was least suspicious about and invited them to a focus group. I was especially conscious that this approach ushered in a significant risk of my own biases influencing the recruitment approach, but wasn’t sure of another path forward. I recorded detailed memos of my decisions and reflected on the decisions with my mentorship team.  In the initial interactions, it became clear that the participants who had signed in to the Zoom meeting were not PICU nurses interested in improving EOL care. Multiple participants pressured me to start the focus group without a quality audiovisual connection and preferred to answer questions in the chat. Participants often shut off their video even when prompted that our conversation would be richer with video on. Finally, when asked the first question of the interview guide (“If during report it became clear that the child you were taking care of would not survive the shift, how would you feel?”), all participants described feelings of fear and that they would attempt to save the child. This, to me, indicated that participants were likely unfamiliar with the nuances of PICU care or EOL care. At that point, my suspicions peaked. I shared that I was concerned about data quality due to the audiovisual and connectivity issues many participants were experiencing, and concluded the focus group.  With the council of my mentorship team and the IRB, we revised our screening procedures to collect hospital email address, collect more verifiable insider information (i.e. number of beds in the PICU you work in), and I cross checked participants free text responses before they could move onto the consent and scheduling process. We continued to use social media recruitment methods, but no longer used public posts and partnered with established community messengers (i.e. nurse influencers we messaged with ahead of time) to recruit our target enrollment population. This improved strategy allowed us to recruit a diverse and legitimate sample of pediatric ICU nurses. |
| --- |
| **Reflection 2 (LL):**  When I began researching the experiences of late-diagnosed Autistic adults in 2015, there was little awareness of this community and few avenues to recruit from this population. For example, autism clinics focused on childhood diagnosis and support, primary care providers often lacked awareness that their patients were Autistic, and few organizations served the needs of the adult community. Yet, what I found online was an active and engaged community of self-advocates coming together through social media to share experiences, build connections, and identify areas of need and potential solutions. I relied on these communities for my new education in autism – to unlearn many of the ableist biases I had been taught, both in society and inherent in my nursing education, and to build a new worldview shaped by neuro-affirming and neuro-inclusive principles. I also relied on these spaces for recruitment, and I carried out both qualitative and mixed methods studies with abundant high-quality data as a result of my connections in these online communities.  However, more recent studies have shown problematic trends in online recruitment. We are currently conducting a qualitative exploration of the health stories of Autistic adults who experience multiple health concerns using a participatory approach, with Autistic community members guiding the research process to be more accessible, respectful, and meaningful to the lives of Autistic people. In building this study, our community team members emphasized the importance of including multiple options for data collection to meet the variety of communication needs of participants. We utilize a multi-modal interviewing approach, in which participants can opt to tell us their story in text: asynchronously through online or mail-in surveys (including personalized follow-up surveys) or synchronously through Zoom chat; or in spoken word or using augmentative and alternative communications: on the phone, on Zoom (with camera on or off), or in person (for local participants only). This approach improves accessibility and affirms diverse ways of communicating.  Within 4 hours of posting our first recruitment notice on social media, nearly 700 “participants” had enrolled in our study and had completed the first stage of the study. As my email box dinged every 2-3 seconds with each new participant, I felt panic rising and immediately closed the study. It was readily apparent that these responses were fraudulent, and our team met to discuss ways to identify and remove surveys completed by “bots.”  Some of the strategies we used, adapted from Pozzar et al. (2020), have included:   - Removing mention of compensation from all recruitment materials (note: compensation is still described on the consent form) - Creating personalized survey links that must be manually generated and sent to each participant after enrolling in the study, rather than using an anonymous link that can be replicated and shared - Adding CAPTCHA to surveys - Adding hidden items to surveys - Monitoring survey completion time for evidence of inattention - Monitoring location for surveys completed outside of the United States (eligibility is limited to those currently in the US) - Developing a protocol to flag surveys meeting these criteria as “suspicious” to be reviewed by our team for quality and authenticity   Even with these changes in place, we encountered participants in Zoom interviews in which it became clear that participants were not intimately aware of the lived experiences of Autistic adults. For example, participants gave overly simplified answers to questions, for example sharing that their experiences were “hard but overall good.” Follow-up questions led to circular answers, with participants essentially restating their stance without additional details, examples, or evidence of thoughtful reflection. These participants followed similar patterns in email addresses, IP addresses, and demographic details including race, gender, and geographic location, as well as similar accents and writing patterns in emails with the research team.  Our team grappled with ways to ensure inclusive data collection methods while still ensuring the integrity of our data. Given that many forms of communication can be disabling for Autistic people (Howard & Sedgewick, 2021), we felt strongly that we could not rely on spoken communication or require a camera on as a means of confirming the legitimacy of our participants. As our team discussed what to do with these responses, our decisions felt particularly problematic since our sample was largely white and these participants represented a racial minority group otherwise missing from our sample. This led to much discussion among our team about the potential for unintentional bias in excluding these accounts. Yet, our team also noted that each of these stories reflected a much more positive view of health experiences than the rest of the sample, which did not align with our understanding of intersectionality and minority health experiences in general. Ultimately, we made the decision to exclude these accounts from analysis.  Overall, our experiences with fraudulent data have been frustrating and deeply saddening. The adult Autistic community remains largely invisible in healthcare, and we know that online recruitment is an important avenue for us to center their voices. Our team has spent an enormous amount of time revising our strategy, submitting modifications to our Institutional Review Board, updating our materials, and monitoring our data. I feel angry that we have had to make compromises to accessibility and inclusivity for the sake of mitigating fraudulent data, but I also feel a responsibility to our participants to protect the authenticity of their stories and the integrity of our findings. We remain hopeful that creativity and adaptability will help us balance these needs. |
| **Reflection 3 (EC):**  Our team conducted a qualitative study with adolescents and young adults (AYA) with cancer to understand their experiences and gather their opinions on our newly developed intervention. Since this project was conducted during the COVID-19 pandemic, we decided to recruit participants through online advertisements. The IRB-approved advertisements were shared on social media (e.g., Facebook), ResearchMatch, AYA cancer foundations’ sections for research advertisements, and hospitals' newsletters and listservs. This age group is familiar with cyberspace and digital technologies, and based on the successful recruitment outcomes of previous studies, we expected the recruitment process to be fairly easy and straightforward.  As anticipated, we received many responses from potential participants interested in our study. The advertisement contained a link to a REDCap survey, which included screening questions (e.g., age) and briefly asked for their clinical information (e.g., diagnosis, treatment). It then followed up with questions requesting their contact information for outreach. Participants who passed the screening questions and seemed eligible were contacted via email. We conducted a Zoom meeting with these participants, went through an informed consent process, asked them to complete a brief demographic and background information survey, confirmed their eligibility again based on their survey responses, and then conducted a 30 to 60-minute online interview.  To be honest, at the beginning, I had no suspicions, as this is a qualitative study involving interviews. Some literature even recommends including qualitative interview components to prevent inappropriate responses from fraudulent participants. However, during the interviews, I began to suspect that some might not be actual cancer patients. For instance, some reported an age different from what they provided in the screening survey during the demographic survey phase and seemed not to know the primary purpose of the study. These individuals were excluded from the study. As the Principal Investigator (PI), I would say to them, ‘I am so sorry, but it appears you are not eligible for this study. We cannot continue.’ Subsequently, they would ask, ‘Does this mean I cannot receive reimbursement?’ Some refused to answer certain demographic or clinical questions, stating discomfort. As we had informed them that they were not obliged to answer any questions they preferred not to, we could not compel them to do so. During the interviews, however, I discovered that some participants had little to no knowledge about cancer or its treatments. While patients can have varying levels of education or knowledge about cancer, based on my experience, young cancer patients are usually very knowledgeable about their condition and treatment. The level of ignorance exhibited during the interviews was concerning. Some simply refused to answer most cancer-specific questions or responded inappropriately; for example, when asked about the perceived benefits of the psychosocial support intervention, they would say, ‘this would help my nutrition,’ without being able to explain their reasoning. At times, they just giggled and did not focus on the interview.  Initially, I was unsure what was happening, but it became clear that these individuals could not be genuine cancer patients. I was seriously concerned about the integrity of my data. I stopped the recruitment process and reported the issue to the IRB. As I had already completed the interview with this person, I had to consider whether I could and should remove this interview record from my data. Nevertheless, I still had to provide reimbursement to this participant since it was not specified in the informed consent form that reimbursement was contingent upon confirmed eligibility, and the interview had been completed. It feels wrong because this decision may inadvertently encourage fraudulent activity, potentially repeating in other research projects. After extensive discussions with my mentor, submitting a deviation report to the IRB, and considering potential solutions, we decided to remove this data from our dataset. We also submitted an IRB amendment and added a statement to the informed consent form indicating that participants could be excluded from the study after enrollment if they are identified as ineligible or not cooperating with the research activities. We identified another recruitment area and posted our advertisement again to complete our study.  Even after making these adjustments, the biggest issue was the trauma I experienced as a qualitative researcher. As an interviewer, I have always believed in a trustful relationship between interviewer and interviewees. However, this incident made me question every interview, constantly assessing whether the participant was a genuine cancer patient. It was a painful experience not to be able to trust my participants and always having to judge the veracity of their statements. I was unprepared for such events and lacked the skills to make judgments on this issue and to communicate clearly with those who were suspicious. To verify the authenticity of ‘cancer patients,’ I sometimes included questions about their experiences; however, I also felt uncomfortable probing into their personal lives not for my research purposes, but to judge the authenticity of their condition. I typically enjoy qualitative interviews for the opportunity to listen to their experiences and learn from their unique stories, but this study turned into a nightmare for me as a qualitative researcher.  Even with these challenges, I still believe in the potential of online recruitment. For my subsequent study, I conducted another online recruitment, but I now avoid posting my research advertisements in public areas, including social media. The struggle to find participants, especially in healthcare research involving human subjects, has intensified since the COVID-19 pandemic. Online recruitment can still be an effective way to reach diverse participants, particularly those who are minorities or living in rural areas. Nevertheless, I wonder whether researchers are fully aware of what is happening in this field and know how to handle such unexpected challenges, including potential fraudulent research participants. These issues can significantly impact the quality of data, the results of analyses, and the knowledge generated. Ignorance of such problems could perpetuate fraudulent activities and pose further challenges to other research projects. Clear guidelines are needed to prevent such issues and, moreover, to address them when they arise. |
