# Supplementary material for: Striking a Balance: Mitigating Fraud While Ensuring Equity in Online Qualitative Research Recruitment
Source: J Med Internet Res. 2025 Aug 27;27:e68393. doi: 10.2196/68393 (PMC12423605; doi:10.2196/68393)
Supplement: Multimedia Appendix 2 [file jmir_v27i1e68393_app2.docx]

## Appendix 2. Fraud moderation strategies & inclusivity principles

## **Phase 1: Study Design, Preparation & Reflection**

| **Question** | **Potential Threats & Rationale** | **Strategies to Address** | **Ethical & Inclusivity Consideration** |
| --- | --- | --- | --- |
| [Reflection – Population] How well do you understand the nuances, risks, and realities of your target population, including the potential for both authentic and deceptive participation in online research? [1-7] | - Misinterpretation of narratives due to lack of contextual understanding  - Overlooking deception embedded in plausible stories  - Mislabeling atypical but legitimate responses as fraudulent  - Participants altering personal info to protect themselves from scams  - Overreliance on narrow fraud detection tools not suited for diverse populations  - A lack of understanding of the target population could lead to overtrust in participant authenticity or inadequate real-time monitoring | - Reflect on potential narrative patterns and their underlying intent during study design  - Train research team to critically assess authenticity without bias  - Use screening criteria appropriate for the population’s cognitive and literacy levels  - Offer a question that aligns with population-specific knowledge or experience  - Involve the community in developing fraud prevention measures | - Avoid assumptions based on culture, language, immigration status, or communication style  - Do not penalize participants who alter info for privacy protection  - Protect participant dignity while remaining alert to deception  - Maintain awareness of imposters without stereotyping |
| [Self-reflection] What is your level of preparedness—both methodologically and emotionally—for managing complex ethical and data integrity issues in qualitative research, including the risk of encountering fraudulent participants? [4, 6-8] | - Emotional distress, frustration, or trauma from engaging with fraudulent participants  - Lack of expertise may result in mishandling deceptive data or misinterpreting participant behavior  - Ethical challenges may be overlooked or inadequately addressed without adequate reflection and protocol | - Build emotional support systems for researchers encountering fraud-related challenges  - Develop clear, systematic protocols to guide responses to suspected fraud  - Incorporate planning for researcher burden into study logistics  - Encourage research team self-reflection to gauge preparedness and skill gaps | - Acknowledge ethical complexities without making assumptions based on language, culture, or communication style  - Ensure ethical reflection is embedded in training and protocol development  - Approach suspicions of fraud carefully, maintaining respect and dignity for participants |
| [Protocol Preparation – General] What specific strategies are you incorporating into your study protocol to detect and prevent fraudulent participation in online qualitative research? [3-11] | - Deceptive data may be embedded in seemingly plausible narratives  - Fraud can emerge at any phase of the study, and evolve over time  - Researchers may lack clear procedures or emotional readiness to respond  - A lack of clear protocol could lead to misidentifying protective behavior (e.g., altered information to protect privacy) as fraud, the risk of vague or generalized data from fraudulent interviewees, and overtrust in participant authenticity or inadequate real-time monitoring. | - Reflect on narrative types and their intent during study design  - Conduct a pilot study to identify risks in recruitment  - Create standardized, team-wide fraud protocols including scripts and classification systems  - Use authentication tools to verify human participants  - Plan interviews to include repeated or reframed questions for consistency checks  - Employ real-time monitoring dashboards with dedicated team access  - Include fraud probability scoring tools for screening  - Collaborate with IRB to ensure ethical oversight of fraud protocols | - Avoid labeling participants as fraudulent solely based on privacy-protecting behavior  - Balance data protection with minimal burden and privacy risks  - Ensure transparency during consent about fraud protocols without discouraging genuine participation  - Avoid stereotyping or over-policing certain groups  - Uphold dignity while preparing to detect imposters |
| [Protocol – Participant Engagement] Have you integrated engagement strategies into your study design to both build trust with participants and better understand the population in ways that help detect and respond to potential fraud? [1, 2, 12, 13] | - Difficulty identifying fraud without in-depth understanding of population-specific behaviors  - Increased exposure to fraud when recruitment is not community-based  - Efforts to reduce participant burden may unintentionally ease entry for fraudulent participants  - Informing participants about fraud risks might cause anxiety or deter valid participation | - Collaborate with relevant community organizations to recruit more trustworthy and contextually informed participants  - Involve community members and researchers with lived experience to co-design tools and identify suspicious responses  - Include participant voices when designing strategies to reduce burden while still protecting data integrity  - If informing participants about potential fraud, provide clear rationale and frame communication to avoid fear or stigma | - Be transparent without compromising participant safety or dignity  - Respect the contributions of community members and co-researchers, particularly from underrepresented or neurodiverse groups  - Ensure engagement approaches are culturally and cognitively appropriate  - Avoid language or procedures that may exclude or alienate valid participants |
| [Protocol – Commitment] Has your research team allocated adequate time, funding, and staffing to support participant verification and continuous monitoring processes throughout the study? [3, 11, 14] | - Underestimating the time, funds, or effort required for proper fraud monitoring  - Gaps in real-time monitoring can allow fraudulent participants to affect data quality  - Verification methods (e.g., phone checks) may increase staff burden and inadvertently exclude legitimate participants | - Allocate dedicated funds and time for verification procedures and system setup  - Use live dashboards and internal communication tools to enable ongoing data monitoring across all phases  - Consider using phone verification systems with protocols in place to assess and interpret use of virtual numbers cautiously  - Prepare for potential scenarios that may require quick response to suspected fraud | - Ensure verification methods are proportionate and respectful of participants’ privacy  - Avoid disqualifying participants solely due to the use of privacy-protective tools like virtual numbers  - Maintain transparency about how verification data is used and stored  - Balance efficient fraud detection with respect for diverse communication and technology use patterns |
| [Protocol – Team Communication] Has your research team developed a communication plan that supports timely, coordinated responses to unexpected issues, including suspected fraud, during the study? [3, 6, 13] | - Lack of real-time communication may delay responses to suspicious activity or data anomalies  - Inconsistencies in team response due to unclear or unstandardized processes  - Interviewers may lack training to interpret or act on early signs of fraud | - Establish a clear, standardized communication plan with protocols for unexpected events  - Schedule regular team meetings to review progress and share observations  - Train interviewers to identify and document potential fraud indicators using both reflective judgment and systematic reasoning  - Encourage a culture of open, nonjudgmental communication within the team | - Ensure communication procedures respect the confidentiality of participant information  - Support interviewers in discussing uncertain or ethically sensitive cases without fear of blame  - Standardize documentation practices in a way that does not encourage profiling or stereotyping of participants  - Build inclusive team norms where all members, regardless of role, can raise concerns |
| [Protocol – Technological Support] Are you prepared to use technological tools to detect and prevent fraudulent participation, and do you understand both their capabilities and limitations in the context of your study population? [1-4, 7-11, 14-16] | - Bots or fake participants may bypass weak authentication systems  - IP address data may be misleading due to shared/public networks or intentional masking  - Over-reliance on automated tools may falsely exclude participants from marginalized groups trying to protect their privacy  - Technical limitations in survey platforms may prevent consistent application of verification measures | - Use CAPTCHA and bot-detection tools to filter out non-human responses  - Cross-check IP addresses, time zones, and survey metadata (e.g., completion times) to spot inconsistencies  - Consult IT experts to monitor activity logs and support real-time verification  - Develop a secure study website with login or email verification  - Set up automated and manual screening systems and assign team members to manage them  - Include verification questions and repeat key items for consistency checks  - Consider asking for formal or organizational emails if appropriate for the population | - Avoid excluding participants who use privacy tools or non-traditional internet access, especially in marginalized or international groups  - Ensure any use of tracking tools (e.g., IP monitoring) is clearly explained in the consent process and approved by the IRB  - Avoid assumptions based solely on technical data; combine with contextual interpretation  - Be transparent about what data is collected and how it will be used and stored securely |
| [Protocol – Mentor and System Support] Have you communicated with mentors, IRBs, funders, and other support systems about the risk of fraudulent participation and involved them in creating strategies to manage it? [4, 8, 15, 16] | - Lack of awareness or preparedness among IRBs and sponsors may delay appropriate responses to fraud  - Absence of guidance from mentors or experienced researchers may limit capacity to recognize and manage suspicious patterns  - Researchers may feel isolated or overwhelmed by the emotional impact of fraud without institutional or mentor support | - Discuss the potential for fraud early with IRBs and advocate for their engagement in supporting research integrity  - Consult mentors and experienced qualitative researchers to develop detection strategies and interpret data authenticity  - Involve legal experts, IRBs, and funders in creating appropriate action plans and protocols, especially for handling data or participant disputes  - Build a system of institutional support for the emotional and ethical burden fraud may place on researchers | - Promote shared responsibility among research systems while maintaining respect for participant rights and privacy  - Ensure any actions related to fraud management align with ethical research standards and are not punitive or overly surveillance-based  - Avoid delegating fraud detection solely to researchers without institutional backing and clear guidance |
| [Recruitment Preparation – Channel] Is your recruitment strategy designed to minimize exposure to fraudulent participants by prioritizing closed or verified channels? [1, 4, 5, 8, 10, 12, 14, 15] | - Public recruitment (e.g., open social media posts) increases the risk of attracting fraudulent participants seeking incentives  - Snowball sampling, while effective, can extend invitations to deceptive individuals  - Verified accounts or hashtags may still be used deceptively or manipulated - Closed recruitment may slow down enrollment or exclude those without access to specific networks | - Prioritize closed or targeted recruitment channels such as professional organizations, invite-only processes, or word of mouth  - Use individualized or separate survey links for different recruitment sources to control and disable links if fraud is detected  - For social media recruitment, apply targeted hashtags and consider validated platforms when possible  - Collaborate with relevant organizations to access verified participant pools | - Ensure closed recruitment methods do not unfairly exclude hard-to-reach populations  - Be transparent about recruitment processes and limitations to participants  - Weigh the trade-off between data quality and accessibility when choosing between public and closed recruitment  - Use inclusive messaging in targeted recruitment efforts to promote equitable participation |
| [Recruitment Preparation – Eligibility] Does your recruitment material balance the need to communicate eligibility criteria with the need to protect the study from fraudulent manipulation? [1, 4, 8, 10, 12, 14] | - Fraudulent participants may tailor responses to match advertised eligibility criteria  - Publicly disclosing full eligibility details allows repeat attempts to game the screening process  - Overly vague or secretive eligibility information may discourage or confuse legitimate participants | - Limit eligibility details in advertisements to essential information only  - Omit sensitive screening criteria that could be manipulated  - Use individualized links for screening to monitor and control access  - Design screening questions to detect repeat attempts or inconsistent responses | - Clearly communicate necessary expectations (e.g., camera use) without over-disclosing criteria  - Avoid creating barriers for legitimate participants who may have questions about eligibility  - Ensure the consent process includes transparent explanations once eligibility is confirmed  - Maintain fairness by applying consistent and ethically justified screening methods |
| [Recruitment Preparation – Incentive Methods and Delivery] What type of incentive will be used, and how will it be delivered in a way that balances fraud prevention, participant accessibility, and ethical compensation? [2-4, 7, 8, 11, 13, 15] | - Monetary incentives may attract fraudulent participants or organized scam groups  - Automated delivery methods may prevent fraud detection and increase risk  - Requesting physical addresses or bank details may raise privacy concerns or exclude vulnerable participants  - Participants may alter screening responses to access high-value incentives | - Offer non-transferable or country-restricted digital vouchers  - Consider using physical mail to verify location, with discreet packaging to protect privacy  - Use manual delivery processes to detect irregular patterns and allow fraud screening  - Prorate compensation based on verified participation level and clarify this in consent  - Explore alternative or supplementary incentives (e.g., training, book giveaways), or engage participants in the research process | - Do not eliminate compensation altogether; this risks excluding under-resourced participants and failing to respect their contributions  - Ensure compensation methods are inclusive for those without stable housing, formal email, or bank accounts  - Clearly communicate compensation conditions and rationale in accessible language  - Avoid requiring unnecessary personal information that could compromise participant safety or trust |
| [Recruitment Preparation – Incentive Information] Is your recruitment material designed to limit the visibility of financial incentives while still meeting ethical standards of transparency? [1, 2, 4, 8, 12-15] | - Publicly advertising financial incentives can attract fraudulent individuals or bots  - Automated systems may scrape social media posts with hashtags or recognizable monetary terms  - Withholding compensation details too long may deter genuine participants or raise ethical concerns about informed consent | - Avoid disclosing specific monetary values in public recruitment materials  - Use alternative terms for incentives rather than "payment" or "gift card"  - Do not use the dollar sign ($) or compensation-related hashtags on public platforms  - Disclose full incentive details only during the screening or consent phase  - Consider spelling out email addresses to avoid scraping by bots | - Ensure participants receive full information about incentives before formal consent, even if not included in the initial recruitment materials  - Frame incentive information in ways that minimize scam risk while respecting participants’ right to know  - Be cautious not to create perceived deception or ambiguity for legitimate participants  - Provide a clear rationale for delayed disclosure, if used, in consent or FAQ documents |
| [Recruitment Preparation – Verification] Are you using additional screening or verification tools to confirm participant authenticity and reduce the risk of fraudulent participation? [1-4, 11-14] | - Public access to screening surveys without controls can lead to bot or scammer activity  - Fraudulent participants may manipulate their identity or IP address  - Verification services may yield inaccurate results or exclude marginalized populations protecting their privacy | - Use CAPTCHA tools or verification systems to confirm human participation  - Include hidden or logic-based questions to detect automated responses  - Route participants through an intermediate step (e.g., email contact or website form) before providing access to surveys  - Verify IP addresses and location data (latitude/longitude) to confirm geographic eligibility  - Consider video calls or requiring formal email addresses for identity confirmation in high-risk studies | - Recognize that participants from vulnerable groups may conceal identifying information for safety  - Avoid overly invasive verification procedures that could compromise trust or participation  - Clearly explain why certain verification steps are in place and how privacy will be protected  - Tailor verification approaches to population characteristics and tech access |
| [Recruitment Preparation – Informed Consent Form] Does your consent form clearly communicate participation requirements, technological expectations, ineligibility criteria, and how potential fraudulent activity will be handled? [4, 7, 9, 10, 13, 14, 16] | - Lack of clarity in consent forms can lead to misunderstandings about participant responsibilities or eligibility  - Fraudulent participants may exploit vague or incomplete consent information  - Participants may be surprised or distressed by verification steps if not disclosed in advance | - Clearly state all study requirements in the consent form, including use of video during interviews, if applicable  - Describe technological expectations and verification procedures (e.g., use of IP address, video confirmation), and distinguish these from study-related data  - Clarify that participation alone does not guarantee compensation and that ineligible or fraudulent cases will not be compensated  - If prorating incentives, explain the rationale and structure transparently | - Ensure participants understand which data will be used for identity verification and why  - Present requirements and fraud policies in accessible, non-threatening language to maintain rapport  - Allow participants flexibility where possible (e.g., partial camera use), and explain the purpose of each requirement  - Secure IRB approval for all verification-related consent content |
| [Recruitment Preparation – IRB Confirmation] Have you included all fraud-related procedures and materials in your IRB submission, and have you discussed potential fraud scenarios and response strategies with your IRB? [3, 4, 7, 8, 10, 15] | - IRBs may not proactively address research fraud, focusing primarily on participant protection  - Lack of IRB-approved language and procedures can prevent researchers from implementing fraud screening steps  - Unexpected fraud-related issues may require protocol amendments, delaying study progress if not anticipated | - Include detailed fraud prevention procedures (e.g., verification steps, eligibility checks, repetitive questions) in the IRB application  - Collaborate with the IRB early to co-develop scripts, screening templates, and data handling protocols  - Clearly distinguish which participant data will be used for verification vs. research purposes and describe this in IRB documents and consent forms  - Build flexibility into your study timeline to allow for IRB amendments as new risks emerge | - Ensure IRB approval for all fraud-related procedures to uphold participant rights and data ethics  - Transparently communicate the purpose of all verification methods to participants in language reviewed by the IRB  - Collaborate with IRBs to develop evolving guidance that balances participant protection with research integrity  - Avoid unnecessary invasiveness in verification processes and consider participant fatigue and privacy |
| [Systematic Decision Making System] Have you established a systematic and flexible decision-making system to identify, assess, and respond to potential fraud across all phases of your study? [1, 4-6, 11] | - Lack of standardized systems may lead to inconsistent or biased fraud detection  - Overly rigid screening systems (e.g., requiring legal ID) may exclude vulnerable populations or raise ethical issues  - Failure to anticipate fraud at all study phases can compromise data quality and response time | - Develop and document a judgment protocol tailored to your population and study context  - Reference diverse, field-specific approaches from the literature to create a flexible framework  - Establish clear criteria for identifying suspicious data while considering population-specific characteristics  - Integrate monitoring tools (e.g., live dashboard) and build a response plan for emerging fraud scenarios  - Use collaborative decision-making methods (e.g., independent team review, discussion of inconsistencies, fraud probability scores) to reduce bias | - Avoid overgeneralized or invasive screening tools that may disproportionately affect marginalized or sensitive populations  - Make decision-making protocols transparent and include ethical justification for each step  - Involve multiple team members to prevent individual bias and promote fair assessment  - Ensure participant dignity and confidentiality are upheld even during fraud screening |

**Phase 2: Recruitment & Screening**

| **Question** | **Potential Threats & Rationale** | **Strategies to Address** | **Ethical & Inclusivity Consideration** |
| --- | --- | --- | --- |
| [Self-reflection & Preparation for Recruitment and Screening] Before beginning recruitment, do you feel prepared with the necessary protocols, support systems, and ethical awareness to handle both expected and unexpected challenges, including fraud? [4, 6-8, 11] | - Researchers may experience emotional distress or burnout when dealing with fraudulent participants  - Lack of clear screening protocols may lead to inconsistent responses to suspicious data  - Researchers may unintentionally stereotype or exclude legitimate participants without careful reflection | - Establish emotional and institutional support systems  - Use reflexive practices and research memos to support thoughtful and ethical decision-making  - Prepare both automatic and manual systems for fraud detection depending on the context and available resources  - Account for researcher burden in the study timeline and resource planning  - Build rapport while remaining alert, and have clear protocols for unexpected events | - Uphold participant dignity by avoiding assumptions based on language, immigration status, or communication style  - Create screening processes that protect both participants’ privacy and data integrity  - Promote ongoing researcher reflection to avoid biases and support inclusivity throughout recruitment |
| [Participant Validation – 1st Check: Sudden Surges in Interest] Do you have systems in place to detect and respond to sudden surges in participant interest, which may indicate potential fraudulent activity? [4, 6, 12, 16] | - A sudden surge in responses may suggest coordinated fraudulent attempts or bot activity  - Suspicious patterns may include odd submission hours, unusually fast completion times, or similar email formats | - Monitor participant interest continuously, especially after launching new recruitment methods  - Flag large influxes of responses within short timeframes, especially during unusual hours or from claimed local participants in inconsistent time zones  - Use authentication tools (e.g., IP tracking, time zone checks) to verify location data  - Trigger additional verification steps when a surge is detected | - Carefully evaluate suspicious patterns without making assumptions about participants’ identities or backgrounds  - Be transparent about monitoring in consent documentation, especially if location or time data will be used  - Avoid excluding valid participants who may submit late-night responses due to work schedules or time zone differences  - Consider context-specific flexibility when determining what counts as a red flag |
| [Participant Validation – 1st Check: Similar Trends in Personal Information (Including Email Addresses)] Are you reviewing participant email addresses and personal information formats for suspicious patterns while remaining cautious not to exclude legitimate individuals? [4, 6, 8, 12-14] | - Fraudulent participants may use similar or repetitive email formats (e.g., all Gmail accounts with similar format such as name+number@gmail.com, unusual strings)  - Poor grammar, overly brief responses, and identical or suspicious answer structures may indicate fraud  - Participants may share the same accent or similar communication styles, which could raise suspicion but also risk bias  - Some genuine participants may use privacy-preserving or nontraditional email formats | - Monitor and log email domain types and structural patterns across submissions  - Cross-check for duplicate names, email addresses, or phone numbers  - Compare suspicious formats to patterns reported in prior literature  - Use email formatting as one factor within a broader fraud detection system rather than a sole exclusion criterion | - Avoid making decisions solely based on email domains or language style, especially for global or multilingual studies  - Disclose to participants, when appropriate, what types of contact information will be reviewed and why  - Use patterns to guide verification, not to automatically exclude—build in space for case-by-case assessment  - Be sensitive to privacy needs and the possibility of shared access (e.g., family accounts, work domains) |
| [Participant Validation – 1st Check: Geographical Discrepancies] Are you monitoring IP addresses, time zones, and submission patterns for discrepancies that may suggest fraudulent participation? [1, 2, 4, 6-8, 10, 13, 15, 16] | - IP addresses and timestamps may reveal discrepancies between claimed and actual geographic location  - Surveys submitted at odd hours or in rapid succession may indicate participation from different time zones or coordinated fraud  - Participants may use VPNs, public computers, or shared networks, limiting the accuracy of geographic validation | - Use IP tracking and time zone checks (e.g., via email timestamps or scheduling tools like Calendly) to compare claimed vs. actual locations  - Log response timestamps and monitor for unusual patterns in submission timing (e.g., night submissions for local participants)  - Use CAPTCHA, completion time data, and geolocation cross-checks where possible  - Work with IT specialists to analyze patterns and flag suspicious cases for review  - Secure IRB approval for the use of IP and location data | - Be cautious not to penalize participants using public networks, VPNs, or accessing from shared devices  - Avoid assuming deception based solely on timing or location mismatches; use as a prompt for deeper verification rather than automatic exclusion  - Be transparent in the consent form about any data collected for verification purposes  - Balance privacy protection with the need for data integrity, ensuring all methods are ethically reviewed and justified |
| [Participant Validation – 1st Check: Screening Survey Response] Are screening survey responses being reviewed for unusually short completion times, vague or inappropriate content, and consistency with later data? [2, 4, 6-8, 12] | - Fraudulent participants may complete screening surveys too quickly  - Responses may appear vague, generic, or inauthentic—especially open-ended questions  - Follow-up responses (e.g., interviews) may not align with screening data, suggesting inconsistency or deception  - Extremely fast replies to researcher outreach may also be suspicious | - Monitor and log completion time for each screening survey, flagging those with unusually short durations  - Review open-ended responses for appropriateness, relevance, and specificity  - Cross-check screening survey data with interview or follow-up responses for consistency  - Be cautious of patterns in communication that are overly scripted or fast across multiple participants | - Avoid relying solely on completion time as a fraud indicator; consider it alongside other contextual factors  - Ensure open-ended questions are designed to be inclusive and do not require excessive detail that may burden legitimate participants  - Remain open to alternative forms of valid expression, particularly for participants with language or cognitive differences  - Clearly communicate what types of responses are needed and why, while preserving participant dignity and comfort |
| [Participant Validation – 1st Check: High Interest in Incentives] Are you monitoring participant communications for signs of unusually high or disproportionate interest in monetary incentives? [8] | - Fraudulent participants may display excessive focus on financial compensation rather than study details or goals  - High incentive focus may signal intention to manipulate eligibility or participate multiple times. | - Observe participant communication tone—flag inquiries overly centered on payment rather than study content  - Use standardized, non-detailed language about incentives in early interactions to avoid attracting scammers  - Include eligibility verification steps before disbursing incentives to all participants | - Avoid assuming that interest in compensation always indicates fraud; consider socioeconomic context  - Maintain equitable access to compensation while protecting study integrity  - Frame compensation as appreciation for time and contributions to avoid creating a transactional tone |
| [Participant Validation – 1st Check: Open-ended Questions] Are open-ended screening questions used to evaluate participant authenticity through response quality, consistency, and population-specific knowledge? [1, 2, 4, 7-9, 12-15] | - Fraudulent participants may provide vague, inappropriate, or illogical open-ended responses  - Omission of open-ended responses or repeated inconsistencies may indicate inauthentic engagement  - Some may fabricate plausible answers or search for correct responses online  - Overly complex or word-count-based questions may burden or exclude participants with cognitive or language differences | - Use open-ended screening questions that assess topic-specific knowledge or lived experience  - Include verification and attention-check questions, and repeat key concepts to identify inconsistencies  - Design flagging criteria based on response quality, not just completeness  - Consider including objective or scenario-based questions to complement open-ended items | - Ensure open-ended questions are culturally and cognitively appropriate to avoid unintentional exclusion  - Provide IRB-approved rationale for screening questions in the consent form  - Avoid rigid expectations about how “authentic” answers must sound, especially when working with neurodiverse or linguistically diverse participants  - Use open-ended responses to prompt deeper exploration, not automatic disqualification |
| [Participant Validation – 1st Check: Identity Verification] Are you implementing a clear and ethical identity verification process before scheduling interviews to confirm participant eligibility and reduce fraud? [1, 3, 5, 8-16] | - Fraudulent participants may create multiple identities or fabricate eligibility  - Email-only screening may not be sufficient to detect deception  - Legal ID or sensitive document requests may exclude or distress vulnerable participants  - Overly strict identity checks may lead to unintentional exclusion, especially for autistic or privacy-conscious individuals | - Incorporate layered verification steps, such as pre-interview phone or video screening to assess response consistency and rapport  - Compare demographic data from the screening survey with verbal responses in secondary checks  - Consider using non-invasive tools like formal emails, CAPTCHA, time zone, and IP address checks  - If needed and IRB-approved, use supporting materials (e.g., diagnosis form, professional profiles), while clearly explaining their purpose and securing consent | - Avoid mandatory legal ID requests unless justified and IRB-approved  - Ensure identity verification procedures are proportionate, sensitive, and clearly communicated in the consent process  - Offer flexible verification methods to accommodate diverse participant needs  - Use person-led screening as an opportunity to build rapport, not just assess authenticity  - Balance fraud prevention with respect for privacy and inclusivity, especially when handling sensitive data |
| [Participant Validation – 1st Check: Video Confirmation] Do you request participants to briefly turn on their cameras before or at the start of the interview to support identity verification, while clearly explaining the purpose and providing flexibility? [1, 4, 6, 8, 10, 12-14, 16] | - Fraudulent participants may refuse or avoid using video, limiting identity confirmation  - Camera refusal may also stem from legitimate privacy concerns, sensory sensitivities, or technical issues  - Inconsistencies in camera use across screening and interview sessions may indicate impersonation | - Request brief video confirmation before interviews or focus groups as part of the verification process  - Include camera expectations clearly in the study protocol and informed consent  - Offer flexible options such as turning on the camera only briefly or allowing blurred backgrounds  - Use video as one of multiple verification tools, not the sole determinant of eligibility | - Respect participant autonomy by clearly explaining the purpose of camera use and allowing opt-out when justified, while also keeping in mind that citing technological issues has been known as a strategy used by fraudulent participants to avoid suspicion.  - Include accommodations for participants with disabilities, privacy needs, or technological limitations  - Avoid overinterpreting camera use as proof of authenticity—balance it with other contextual and behavioral cues  - Build rapport through the video interaction while maintaining participant comfort and trust |
| [Participant Validation – 1st Check: Recruitment Routes] Are you documenting and monitoring how participants were recruited and which channels they used to access the study? [14-16] | - High-risk recruitment platforms (e.g., public social media) may lead to increased exposure to fraudulent participants  - Lack of documentation may prevent researchers from identifying patterns tied to suspicious responses  - Relying on a single recruitment source can obscure fraud detection if that source is compromised | - Track and log the recruitment source for each participant (e.g., platform, referral, organization)  - Review recruitment trends to identify potential spikes from specific platforms  - Use different survey links for each recruitment route to enable source-specific monitoring and rapid disabling if needed | - Avoid overgeneralizing risks tied to particular recruitment platforms; contextualize findings before making exclusion decisions  - Ensure participants are informed that recruitment sources are tracked for quality assurance (if applicable)  - Use recruitment tracking data to improve inclusive and secure outreach strategies over time in future studies |
| [Training for Recruitment and Screening] Are interviewers adequately trained to detect and respond to potential indicators of fraudulent participation during recruitment and screening? [6] | - Untrained interviewers may overlook red flags or inconsistencies  - Over-reliance on instinct without structured guidance may lead to bias or misclassification  - Failure to document concerns could hinder team coordination and protocol development | - Train interviewers to use both reflective judgment and systematic observation to identify potential fraud  - Provide examples of red flags and appropriate follow-up actions as part of interviewer onboarding  - Encourage consistent documentation of suspicious behaviors and unclear responses for team-based review | - Ensure training includes guidance on avoiding assumptions based on communication style, language, or cultural differences  - Promote a nonjudgmental, inquisitive approach that balances vigilance with empathy  - Clarify that observations are part of a larger verification system—not the sole basis for exclusion |
| [Participant Validation – 1^st^ Check: Privacy Protection] Are privacy protections in place for any additional personal or identifying information collected during participant screening and validation? [4, 5, 15] | - Collecting personal or identifying information may compromise participant privacy or deter participation  - Lack of transparency about why and how identity-related data is collected could erode trust  - Excessively strict verification steps may exclude legitimate participants | - Reassess demographic or identifying information during screening to detect fraud, with IRB approval  - Develop a clear plan for protecting collected identity-related data, including secure storage and retention timelines  - Clearly explain the purpose of any verification steps and what data will be collected and used | - Ensure that any personal data collection is proportionate, justified, and approved by the IRB  - Respect participant autonomy by offering clear explanations and allowing opt-outs when feasible  - Avoid rigid verification that may exclude marginalized or privacy-conscious individuals  - Build trust by demonstrating responsible data handling practices and limiting unnecessary data collection |
| [Participant Validation – 1st Check: Communication, Cooperation, and Intuition] Are you communicating well with participants? Are you assessing the tone, content, attitude, and consistency of participant communication to detect vague or suspicious patterns that may indicate fraud? [3, 6, 8, 10] | - Fraudulent participants often provide vague, brief, or generic responses in initial communication  - Extremely fast or overly enthusiastic replies without clear motivation may raise red flags  - Difficulty in building rapport or obtaining cooperation may indicate impersonation or misrepresentation  - Relying solely on “intuition” may lead to bias or inconsistent judgment | - Pay attention to the tone and depth of participant emails—flag overly vague or overly scripted responses  - Conduct a secondary, person-led screening (e.g., phone call) to clarify details, assess rapport, and verify information  - Compare email and verbal responses for consistency; document any unusual demographic fit or behaviors for team review  - Use team-based review to validate “gut feelings” with evidence-based criteria | - Avoid excluding participants based solely on language proficiency or writing style; consider accessibility and language differences  - Ensure rapport-building efforts are inclusive and culturally sensitive  - Use a consistent, transparent process for flagging concerns rather than relying on individual intuition alone  - Offer opportunities for clarification before making final eligibility decisions |
| [Systematic Decision Making] Do you have a structured, evidence-based system for making participant validation decisions using multiple cues and collaborative judgment? [1, 6, 7, 11, 15] | - No single indicator is sufficient to confirm fraudulent participation—reliance on one cue may lead to false exclusions  - Screening systems may mistakenly flag legitimate participants, especially those with privacy concerns or communication challenges  - Real-time monitoring of participant behavior is necessary to detect evolving fraud patterns | - Use a multi-indicator approach (e.g., screening response quality, time zone, video check) to assess participant authenticity  - Implement a tiered flagging system (e.g., green/yellow/red) to guide review of suspicious cases  - Conduct regular team discussions and independent reviews when suspicious patterns emerge  - Combine automatic tools (e.g., probability scores, dashboards) with manual review when necessary  - Compare observations to prior research or case examples to guide judgment | - Use a transparent and flexible decision-making process to reduce bias and build trust  - Ensure all flagged participants are reviewed fairly, with attention to unintentional errors, protective behaviors, or diverse communication styles  - Include documentation and justification for decisions to ensure consistency and accountability  - Regularly evaluate and refine the system to improve accuracy and inclusivity over time |

**Phase 3: Data Collection, Analysis, and Dissemination**

| **Question** | **Potential Threats & Rationale** | **Strategies to Address** | **Ethical & Inclusivity Consideration** |
| --- | --- | --- | --- |
| [Ethical Consideration] During data collection and analysis, do you maintain ethical standards by respecting participants’ dignity and protecting their privacy—even when fraudulent behavior is suspected? [6, 7, 10, 11] | - Participants may appear deceptive due to unintentional errors or protective behavior, not malicious intent  - Harsh responses to suspected fraud may violate participant dignity or privacy, especially in sensitive studies  - Full separation of identifying information may limit ability to verify or correct suspicious data | - Link data with pseudonyms or non-sensitive identifiers (e.g., pseudo-profiles) to balance privacy and traceability, even when the data comes from fraudulent participants  - Clearly define what identifying information is necessary, and protect it appropriately throughout screening and data collection  - Create ethical, systematic protocols for how potentially fraudulent data will be reviewed, retained, or discarded  - Maintain judgment-free language and procedures, especially when assessing sensitive or vulnerable participants | - Avoid assumptions based on communication styles, language, immigration status, or cultural differences  - Acknowledge that participants may withhold or alter information for safety reasons—not all inconsistencies signal fraud  - Carefully balance data protection, privacy, and research integrity without overcorrecting or overprotecting non-sensitive data  - Document ethical decisions and involve the research team in any decision to remove or retain questionable data |
| [Training for Data Collection and Analysis] Have interviewers and analysts been trained to recognize and respond to potential indicators of fraudulent behavior during data collection and analysis? [6, 13] | - Interviewers or analysts may overlook subtle signs of fraud without appropriate training  - Sole reliance on intuition may result in inconsistent or biased judgment  - Lack of documentation may lead to poor team coordination or decision-making in ambiguous cases | - Train team members to identify early indicators of fraud using both instinct and structured reasoning  - Provide concrete examples and red flags for detection during training  - Establish a consistent process for documenting observations and concerns during data collection and analysis  - Encourage reflective practices and team discussion when uncertainty arises | - Avoid drawing conclusions based solely on intuition or communication style  - Promote an inclusive lens in identifying suspicious activity to reduce the risk of misjudging culturally or linguistically diverse responses  - Ensure training emphasizes balanced, fair, and respectful approaches to participant engagement and data review |
| [Participant Validation – 2nd Check: Consistency of Information] Are inconsistencies between self-reported information (e.g., in screening surveys) and verbal responses during pre-screening or interviews flagged and followed up? [1, 2, 4, 6, 8, 10, 14] | - Discrepancies between written and verbal responses may indicate fabricated information  - Fraudulent participants may struggle to answer repeated or reframed questions consistently  - Inconsistencies in demographic details or medically relevant information (e.g., disease knowledge, stable traits like height) may suggest misrepresentation  - Not all inconsistencies are due to fraud—nervousness, privacy concerns, or language differences may affect responses | - Incorporate verification questions and ask key items multiple times in different ways during screening and interviews  - Cross-check demographic and disease-specific responses from initial screening with interview answers  - Flag major discrepancies for team discussion and follow-up—not immediate exclusion  - Use questions related to stable facts (e.g., chronic traits or lived experiences) to detect manipulation over time | - Clearly inform participants in the consent form if some questions will be repeated or used for consistency checks  - Avoid assuming deception based on inconsistencies alone—offer opportunities to clarify unclear or contradictory answers  - Ensure that repeated questioning is done respectfully and without unnecessary burden or fatigue, particularly for vulnerable participants  - Be cautious not to overinterpret nervousness or hesitation as evidence of fraud |
| [Participant Validation – 2nd Check: Addressing Suspicious Cases During Recruitment/Screening] Do you have a process for identifying and responding to suspicious cases early in the recruitment and screening processes, including documentation, team communication, and access to support if needed? [8, 12, 13, 16] | - Delayed analysis or lack of structured response plans may allow fraudulent data to accumulate  - Lack of clear procedures for managing suspicious cases may result in inconsistent handling or lost evidence  - Sole reliance on subjective judgment may lead to false positives or misclassification | - Begin preliminary data analysis early and regularly during data collection  - Create a code for “suspicious responses” and document cases using memos or reflexive journals  - Hold regular team meetings to review questionable cases, compare observations, and make collective decisions  - Reflect continuously on participant trustworthiness and flag unclear cases for deeper review  - If applicable, prepare a plan for seeking legal or institutional support in extreme fraud cases | - Ensure documentation of concerns is done respectfully and avoids stigmatizing language  - Allow for clarification or follow-up before determining a case is fraudulent  - Include the possibility of mistaken suspicion in team discussions to reduce bias  - Balance rapid response with careful, fair assessment, especially for participants who may appear “atypical” for non-deceptive reasons |
| [Participant Validation – 3rd Check: Logical Consistency] Are participant responses being assessed for internal logic and relevance to ensure they align with the expected experiences or knowledge of the target population? [8] | - Illogical, irrelevant, or inconsistent responses may indicate the participant is not part of the target population or is fabricating information  - Unusual demographic profiles may suggest misrepresentation, but may also reflect genuine variation | - Evaluate responses for alignment with the topic and lived experience expected from the population  - Flag responses with illogical patterns or demographic inconsistencies for further review—not immediate dismissal  - Train interviewers to listen for gaps in logic or topic knowledge during conversations and to follow up with clarifying questions | - Be cautious not to assume deception solely based on what appears “unusual”  - Avoid relying on normative assumptions about what a “typical” participant should sound like  - Provide opportunities for clarification before labeling responses as fraudulent  - Document the reasoning for flagging inconsistencies to support fair and transparent decision-making |
| [Participant Validation – 3rd Check: Response Quality] Are qualitative responses evaluated for detail, relevance, and participant engagement, and are behaviors like refusal to follow instructions or distraction during the interview noted? [2, 6, 8, 10, 14] | - Fraudulent participants may offer vague, overly brief, or off-topic responses  - Shorter-than-average interview durations and refusal to follow instructions (e.g., not turning on a camera) can signal disengagement or deception  - Some unclear or limited responses may stem from nervousness, privacy concerns, or language barriers—not necessarily fraud | - Manually review qualitative responses for depth, coherence, and alignment with the topic  - Use thick, detailed responses as one marker of genuine participation, while probing brief responses for clarification if necessary, as fraudulent participants could still provide rich narratives by searching online or when discussing common experiences.  - Plan interviews with flexible follow-up prompts to elicit clarity and detail  - Document patterns of uncooperative or disengaged behavior (e.g., repeated refusal to follow basic instructions) for team review | - Avoid penalizing participants who provide brief responses due to anxiety, neurodivergence, or communication preferences  - Provide opportunities to clarify or expand answers before making assumptions about fraud  - Ensure instructions are clearly communicated and reasonable, with flexibility when possible  - Respect participants’ right to privacy while balancing the need for authentic, high-quality data |
| [Participant Validation – 3rd Check: Visual and Auditory Cues] Are visual and auditory cues—such as camera use, background environment, and consistency with disclosed information—being observed and considered during data collection? [6, 14] | - Inconsistent or vague visual/auditory context (e.g., background noise, time of day, appearance) may raise suspicion  - Poor technical quality may hinder rapport and make it harder to verify participant identity  - Participants may deliberately obscure visual cues by blurring or disabling video, or this may stem from legitimate privacy needs | - Observe alignment between verbal claims and visual/auditory cues (e.g., location, age appearance, background setting, time zone)  - Ask participants to briefly turn on their camera for verification, if appropriate and IRB-approved  - Note inconsistencies in auditory context (e.g., weather, environment) that do not match reported information  - Document observations for team-based decision-making, rather than relying on individual judgment alone | - Avoid overinterpreting visual or auditory differences as deception—consider cultural, technological, or personal factors  - Allow participants to explain any apparent inconsistencies (e.g., traveling, shared spaces)  - Respect privacy concerns and technological limitations. When requiring camera use, communicate clearly and consider that legitimate participants may still have valid reasons for being unable to turn on their cameras.  - Use visual/auditory cues as one part of a broader, ethically balanced fraud detection system |
| [Participant Validation – 3rd Check: Addressing Suspicious Cases] Do you have a clear, respectful, and IRB-approved protocol for how to respond if a participant appears suspicious or fraudulent during an interview or data collection? [2, 4, 8, 15] | - Interviewers may feel uncomfortable or unsure how to respond in real-time when suspicions arise  - Ending interviews abruptly or accusing participants may harm trust or dignity, especially if suspicion is unfounded  - Some participants may provide legitimate but unusual information, making judgment difficult | - Pause or end the interview politely, citing technical or scheduling issues if needed  - Develop and use a prepared, IRB-approved script for addressing suspected fraud in real time  - Create a protocol for partial compensation, contingent on IRB approval, in cases of inconclusive suspicion  - Debrief with the research team or PI after any uncertain case before making final decisions | - Treat all participants with dignity and avoid making direct accusations  - Remain open to the possibility that suspicious behavior may be due to privacy needs, neurodiversity, or other factors  - Ensure all procedures for handling fraud are IRB-reviewed and proportionate to the study’s sensitivity  - Be transparent in documentation and decisions, and maintain participant confidentiality regardless of outcome |
| [Self-reflection & Preparation for Data Collection & Analysis] Before beginning data collection, do you feel adequately supported, emotionally prepared, and equipped with the protocols and tools needed to handle potential fraud and its impact on your research process? [4-8, 10] | - Emotional stress, fatigue, and self-doubt from encountering fraud may lead to researcher bias or inappropriate decision-making  - Lack of structured support systems may impair judgment or analysis integrity  - Reflexive insight and transparency are essential for maintaining qualitative rigor | - Use reflective journals, memos, and audit trails to document decisions and emotional responses  - Schedule regular team debriefings to share concerns and support one another  - Prepare emotionally for unexpected incidents by acknowledging researcher vulnerability and planning for institutional or peer support  - Clearly define analytic procedures for handling suspicious data, and document decisions transparently in reports or footnotes | - Recognize that not all inconsistencies indicate deception—cultural, emotional, or contextual factors may shape responses  - Approach data collection with sensitivity, openness, and readiness to adapt  - Uphold participants’ dignity while maintaining high standards of research trustworthiness  - Avoid stereotyping and remain grounded in reflexive, ethical practice when making analytic judgments about suspicious data |
| [Participant Validation – 4th Check: Data Consistency] Are timestamps, IP addresses, and location data reviewed for consistency across data sources, and is the transcription process structured to help detect suspicious patterns? [2, 7, 10, 11, 14] | - Fraudulent participants may provide inconsistent timestamps, IP addresses, or location information across touchpoints  - Unusual patterns in data may go unnoticed without dedicated time for review  - Outsourcing transcription may miss subtle cues indicating potential fraud | - Continuously monitor incoming data using live dashboards and systematic review tools  - Compare timestamps, IP addresses, and stated locations for discrepancies  - Have a consistent individual (e.g., the primary researcher) transcribe all interviews to detect subtle inconsistencies or suspicious trends  - Use follow-up questions or interviews to clarify inconsistencies identified during analysis  - Keep reflexive journals to document decision-making, especially when uncertain | - Clearly inform participants during the consent process if any metadata (e.g., IP addresses) will be collected  - Avoid drawing conclusions from minor inconsistencies alone; triangulate with other indicators  - Ensure transcription practices respect participant confidentiality and data security  - If transcription is internal, provide training or structured guidance to maintain accuracy and ethical sensitivity |
| [Participant Validation – 4th Check: Compensation Interest] Do participants exhibit unusually high or immediate interest in receiving compensation, especially with repeated or similarly worded inquiries? [6, 8] | - Fraudulent participants may display disproportionate interest in compensation, contacting researchers quickly after participation and often using repetitive language  - Such behavior may indicate prioritization of payment over genuine study interest | - Monitor and document communication patterns around compensation (e.g., timing, wording)  - Include a standard delay and a clear timeline for incentive delivery, outlined during the consent process  - Use compensation disbursement methods that allow additional verification (e.g., address confirmation or partial payment when suspicion exists and IRB-approved)  - Compare compensation-related communication with participant behavior during data collection for coherence | - Avoid assuming that interest in compensation always signals deception—financial need is valid and context-dependent  - Clearly communicate expectations for compensation in a transparent but neutral tone during onboarding  - Ensure that compensation policies are equitable, ethically justified, and do not penalize low-income or vulnerable participants  - Treat all post-participation communication respectfully, even when suspicion arises |
| [Participant Validation – 4th Check: Across Case Comparison] Do you observe repeated or unusually similar demographic characteristics, language, voices, or environmental cues across participants who should be unrelated? [6] | - Repeated patterns in participant responses (e.g., identical phrasing, background sounds, or voice tone) may indicate duplicate or coordinated fraudulent participation  - Fraudsters may attempt to enroll multiple times under different identities, especially if incentives are involved | - Compare interviews across participants for similarities in demographics, voice, tone, environment, or content  - Maintain a tracking log of shared characteristics to assist with cross-case validation  - Cross-reference metadata (e.g., IP addresses, email domains) alongside content similarities to strengthen pattern recognition  - Review similar-sounding cases in team meetings for collaborative judgment | - Avoid assuming similarity means fraud without corroborating evidence—some overlaps may be coincidental or culturally relevant  - Ensure that participants are not penalized for sharing characteristics common within a community or population  - Use across-case comparison as part of a larger, cautious fraud detection system rather than an exclusion tool alone  - Continue to protect participant dignity and confidentiality in cases flagged for review |
| [Participant Validation – 4th Check: Team Review] Are potentially fraudulent or suspicious cases systematically reviewed through collaborative team discussions using a shared protocol? [1, 4, 6, 8, 13, 16] | - Individual judgments may be inconsistent or biased if not discussed with the team  - Lack of documentation or shared decision-making may undermine transparency and study trustworthiness - Delayed or unresolved concerns may affect data quality and researcher well-being | - Schedule regular team meetings and debriefs to discuss suspicious cases and share concerns  - Use a flagging system that guides collective review and discussion before decisions are finalized  - Maintain reflexive memos and documentation of team decisions for audit purposes  - Use structured processes, including independent reviews (e.g., co-listening to audio), to reduce individual bias  - Pause data collection when needed and consult with the PI for clarity and support | - Encourage open, respectful discussion across the team, including junior members, to support shared accountability  - Acknowledge disagreements and address them collaboratively and transparently  - Ensure that decisions to exclude or question data are based on cumulative, evidence-informed criteria—not intuition alone  - Protect participant dignity throughout the process, especially when fraud cannot be definitively confirmed |
| [Participant Validation – 4th Check: Participant Engagement] Have you considered using participant engagement strategies—such as member checking, follow-ups, or consultation with community partners—to help assess the authenticity of data? [10, 13, 14] | - Fraudulent participants may avoid follow-up opportunities or fail to engage in post-interview communication  - Lack of community involvement may limit researchers’ ability to recognize inauthentic responses  - In group settings (e.g., focus groups), participants may unknowingly interact with fraudulent individuals, affecting data quality | - Conduct follow-up contact (e.g., brief check-ins or member checking) to confirm participation and gather feedback  - Send participants summaries of findings to foster continued engagement and potentially deter fraudulent involvement  - Collaborate with trusted community partners or representatives of the target population to review questionable patterns in data  - If disclosing the presence of fraud to participants (e.g., in group settings), do so without revealing identities and with IRB guidance | - Ensure that any engagement does not unintentionally expose participant identities or compromise anonymity  - Respect participants’ right to remain anonymous and to decline participation in member checking or follow-up  - Clearly explain the purpose of engagement activities in the consent process to build transparency and trust  - Be cautious when interpreting lack of follow-up engagement, as it may be due to accessibility issues or personal reasons, not necessarily fraud |
| [Participant Validation – 4th Check: Addressing Suspicious Cases] Do you have a respectful, systematic, and IRB-approved protocol in place for managing potentially suspicious cases—including scripted responses, documentation practices, and access to support or legal resources if needed? [7, 8] | - Participants may provide unusual or unexpected responses due to personal, cultural, or emotional factors—not necessarily with fraudulent intent  - Lack of a respectful and consistent protocol may harm participants or create inconsistencies in how cases are handled | - Develop and use a pre-approved, respectful script for pausing or ending interviews when fraud is suspected  - Ensure compensation policies are honored when appropriate, even in cases of suspected fraud, unless clear deception is verified  - Train interviewers to recognize the difference between deception and culturally or contextually different responses  - Establish internal review protocols and access to institutional or legal consultation if necessary | - Treat all participants with respect and avoid assumptions about intent without corroborating evidence  - Preserve participant dignity even when data is deemed unusable  - Ensure procedures for terminating participation are transparent and ethically justified  - Involve the research team and IRB in developing or revising response plans to uphold participant protections |
| [Compensation Distribution] Is participant authenticity verified before compensation is distributed, and are there clear procedures for handling suspicious cases? Do your IRB materials and consent form explain under what conditions compensation will or will not be provided? [15] | - Fraudulent participants may complete data collection solely to receive compensation  - Suspicious payment requests (e.g., non-residential mailing addresses) may go unnoticed without manual review  - Without clear IRB-approved language, researchers may feel obligated to compensate even questionable participants | - Researchers can consider distributing incentives only after verifying participant eligibility and data quality. However, if their IRB approval does not include this requirement, they may still be obligated to provide compensation once data collection is completed—even if they are aware that the case is fraudulent.  - Review suspicious compensation requests manually (e.g., verify mailing addresses or review response quality)  - Include clear IRB-approved language in the protocol and consent form stating that ineligible or fraudulent participants will not receive compensation, even if they complete an interview  - Keep thorough documentation of decisions related to compensation to support transparency and prepare for potential disputes | - Balance fraud prevention with fair compensation for participants who engage in good faith  - Clearly explain compensation policies up front, including conditions under which payment may be withheld  - Consider partial compensation in borderline cases, with justification and IRB approval  - Explore non-monetary forms of engagement (e.g., offering research findings or community resources) while ensuring that participants’ time is valued and fairly acknowledged |
| [Documentation and Reporting – Deceptive Data] Are suspicious or fraudulent cases and their data documented clearly and transparently reported in research outputs, while protecting participant dignity and ensuring IRB and funder communication? [3-5, 8, 13] | - Avoiding discussion of fraudulent participation may harm scientific integrity and lead to repeated errors in future research  - Readers may misinterpret transparency as a study weakness  - Incomplete documentation can hinder team learning and ethical accountability | - Maintain a coding system (e.g., “suspicious response” code) during analysis for clear tracking  - Include transparent notes in research outputs (e.g., footnotes, methodology sections) explaining why certain data were excluded or flagged  - Report issues to IRB and, when appropriate, funders and sponsors  - Keep records of verification efforts, but establish clear policies on data retention length and purpose | - Treat even fraudulent participants as human subjects with dignity and care in how their data is discussed and stored  - Frame reporting of suspicious cases as a contribution to field-wide learning, not a personal or institutional failure  - Clarify in publications that detecting and reporting fraud is part of responsible research practice  - Reflect on the ethical implications of retaining, deleting, or anonymizing deceptive data after study closure |
| [Documentation and Reporting – Lessons Learned] Are the insights gained from dealing with fraudulent participation documented and used to improve future research design, protocols, and training? [3, 4, 6, 7, 10, 11, 15, 16] | - Failure to document and share lessons learned may lead to repeated vulnerabilities across studies  - Without reflection, researcher fatigue and bias may go unaddressed  - Omission of these lessons from academic discourse may hinder research transparency and credibility | - Write transparent summaries of suspicious cases, the steps taken, and their outcomes to inform future research  - Maintain research memos, audit trails, and reflexive journals throughout the process  - Integrate lessons into future IRB applications, protocols, recruitment plans, and staff training  - Use appropriate reporting tools to track and evaluate the emotional and procedural impact of encountering fraud  - Share findings in academic settings to promote field-wide preparedness and integrity | - Ensure transparency does not stigmatize or oversimplify participants’ actions—contextualize experiences with care  - Reflect on and document researcher emotions and experiences to foster ethical reflexivity  - Use documented lessons to strengthen future research while maintaining respectful, equitable approaches to participant engagement |
| [Documentation and Reporting – Detection Strategies] Are the fraud detection strategies, rationale for judgments, and exclusion criteria clearly documented and included in study reports or publications? [1, 3, 8, 11, 13] | - Lack of transparency about how suspicious data were identified and excluded may weaken research credibility and replicability  - Over-disclosure of detection strategies could unintentionally aid future fraudulent participants in avoiding detection  - Transparency in reporting may be misinterpreted as poor data quality or weak study design | - Include a clear, transparent summary in publications detailing what happened, how fraud was identified, and why certain data were excluded  - Describe the detection process and rationale using neutral, nonjudgmental language in methods or limitations sections  - Maintain internal records of fraud detection methods and share with IRBs, funders, and trusted academic audiences  - Consider what to publish publicly vs. what to retain internally to avoid giving fraudsters an advantage | - Frame transparency about fraud detection as a strength, showing ethical diligence rather than weakness  - Use anonymized examples or aggregated patterns rather than detailed “how-to-detect” lists in public documents  - Ensure all disclosures protect participant privacy, regardless of fraud status  - Continue contributing to broader community learning by responsibly reporting challenges and strategies |
| [Collaborating with Community: IRB, Funders, Academic Communities] Are you engaging IRBs, funders, legal experts, and the broader academic community in conversations about fraudulent participation, protocols, and prevention strategies? [1, 3, 4, 8, 12, 13, 15, 16] | - IRBs and funders may not yet have established guidance for research fraud, leaving researchers without institutional support  - Without open academic dialogue, lessons learned from fraudulent cases may be lost or repeated by others  - Lack of collaboration can limit innovation in fraud prevention tools, including ethical use of emerging technologies | - Collaborate proactively with IRBs when developing fraud detection and response protocols, including consent language, scripts, and documentation templates  - Report experiences and suspicious activity to IRBs, funders, and sponsors, especially when data integrity or financial resources may be compromised  - Share experiences and findings transparently with the academic community through publications, presentations, and policy dialogues  - Participate in or initiate cross-disciplinary discussions on the role of technology (e.g., AI) in fraud detection, acknowledging both risks (e.g., fraudulent participants could also use advanced technology) and benefits | - Promote collective responsibility across institutions and disciplines to address and learn from fraudulent participation  - Frame community collaboration as a means of strengthening—not weakening—participant protection and research credibility  - Ensure that transparency does not violate participant confidentiality, especially in small or vulnerable populations  - Advocate for ethical, inclusive, and balanced approaches in developing community-wide guidelines and training tools |

**References**

1. Carey ME, McLean KJ, Chvasta K, de Marchena A, Roux AM. Methods to reduce fraudulent participation and highlight autistic voices in research. Autism;2024:13623613241298037. doi:10.1177/13623613241298037

2. Davies MR, Monssen D, Sharpe H, Allen KL, Simms B, Goldsmith KA, et al. Management of fraudulent participants in online research: Practical recommendations from a randomized controlled feasibility trial. Int J Eat Disord;2023. doi:10.1002/eat.24085

3. Glazer JV, MacDonnell K, Frederick C, Ingersoll K, Ritterband LM. Liar! Liar! Identifying eligibility fraud by applicants in digital health research. Internet Interv;2021;25:100401. doi:10.1016/j.invent.2021.100401

4. McLachlan K, Truffyn EE, Dunleavy B, Linkiewich D, Powell D, Taddio A, et al. Fraudulent participation in psychological research using virtual synchronous interviews: Ethical challenges and potential solutions. Ethics Behav;2024:167-189. doi:10.1080/10508422.2024.2347658

5. Owens LL. Encountering deception in virtual spaces guidelines for virtual ethnography. Front Sociol;2023;8:1163560. doi:10.3389/fsoc.2023.1163560

6. Sansfaçon A, Gravel E, Gelly MA. Dealing with scam in online qualitative research strategies and ethical considerations. Int J Qual Methods;2024;23. doi:10.1177/16094069231224610

7. Wang Y. Do participants lie? Imposter participants in online qualitative research. Qual Res J;2024. doi:10.1108/QRJ-06-2024-0130

8. Mistry K, Merrick S, Cabecinha M, Daniels S, Ragan J, Epstein M, et al. Fraudulent participation in online qualitative studies: Practical recommendations on an emerging phenomenon. Qual Health Res;2024:10497323241288181

9. Mizerek E, Wolf L, Moon MD. Identifying and mitigating fraud when using social media for research recruitment. J Emerg Nurs;2023;49(4):530-3. doi:10.1016/j.jen.2023.04.002

10. Roehl JM, Harland DJ. Imposter participants: Overcoming methodological challenges related to balancing participant privacy with data quality when using online recruitment and data collection. Qual Rep;2022;27(11):2469–2485. doi:10.46743/2160-3715/2022.5475

11. Willis TA, Wright-Hughes A, Skinner C, Farrin AJ, Hartley S, Walwyn R, et al. The detection and management of attempted fraud during an online randomised trial. Trials;2023;24(1):494. doi:10.1186/s13063-023-07517-4

12. Kumarasamy V, Goodfellow N, Ferron EM, Wright AL. Evaluating the problem of fraudulent participants in health care research: Multimethod pilot study. JMIR Form Res;2024;8:e51530. doi:10.2196/51530

13. Pellicano E, Adams D, Crane L, Hollingue C, Allen C, Almendinger K, et al. Letter to the editor: A possible threat to data integrity for online qualitative autism research. Autism;2024;28(3):786-92. doi:10.1177/13623613231174543

14. Wright M, Matheson J, Watson TM, Sproule B, Le Foll B, Brands B. Participant fraud in virtual qualitative substance use research: Recommendations and considerations for detection and prevention based on a case study. Subst Use Misuse;2024;59(8):1261-70. doi:10.1080/10826084.2024.2330892

15. Panicker A, Nurain N, Ibrahim Z, Wang CH, Ha SW, Wu Y, et al. Understanding fraudulence in online qualitative studies: From the researcher’s perspective. Proceedings of the CHI Conference on Human Factors in Computing Systems;2024. p. 1-17. doi:10.1145/3613904.3642182

16. Sefcik JS, Hathaway Z, DiMaria-Ghalili RA. When snowball sampling leads to an avalanche of fraudulent participants in qualitative research. Int J Older People Nurs;2023;18(6):e12572. doi:10.1111/opn.12572
